# Safety and Immunogenicity of a Live Attenuated RSV Vaccine in Healthy RSV-Seronegative Children 5 to 24 Months of Age

**DOI:** 10.1371/journal.pone.0077104

**Published:** 2013-10-29

**Authors:** Elissa Malkin, Ram Yogev, Nazha Abughali, Joseph Sliman, C. Kathy Wang, Fengrong Zuo, Chin-Fen Yang, Mark Eickhoff, Mark T. Esser, Roderick S. Tang, Filip Dubovsky

**Affiliations:** 1 Clinical Development, MedImmune, Gaithersburg, Maryland, United States of America; 2 Pediatric, Adolescent and Maternal HIV Infection, Ann & Robert Lurie Children's Memorial Hospital, Chicago, Illinois, United States of America; 3 Department of Pediatrics, Case Western Reserve University School of Medicine, Cleveland, Ohio, United States of America; 4 Applied Immunology and Microbiology, MedImmune, Mountain View, California, United States of America; 5 Research & Development, MedImmune, Gaithersburg, Maryland, United States of America; 6 Clinical Operations, MedImmune, Gaithersburg, Maryland, United States of America; The George Washington University Medical Center, United States of America

## Abstract

Despite substantial morbidity associated with respiratory syncytial virus (RSV) infection, there is no licensed vaccine. MEDI-559 is a live attenuated intranasal vaccine candidate being developed for prevention of lower respiratory illness due to RSV in young children. This randomized, placebo-controlled study evaluated safety of MEDI-559 in healthy, RSV-seronegative children. MEDI-559 or placebo was administered on 3 occasions, 2 months apart. Primary safety was based on solicited symptoms (SSs) and adverse events (AEs) collected for 28 days after each dose. Nasal wash samples were collected 3 times after each dose (days 7-10, 12-18, 28-34) and at sick visits. Serum was collected for measuring antibody immune responses to RSV prior to first vaccination and 28 days post final dose. Long-term safety was monitored for 365 days from first dose. SSs were mild and frequent (MEDI-559 84%; placebo 91%); most common SSs were runny/stuffy nose, cough, and irritability/fussiness. AEs occurred in 67% MEDI-559 and 57% placebo recipients: most common AE was upper respiratory tract infection (MEDI-559 35%; placebo 23%). Higher incidence of medically attended lower respiratory illness within 28 days after dosing occurred in the MEDI-559 arm compared to placebo (none associated with vaccine virus shedding). There was no evidence of enhanced RSV disease. Vaccine virus was detected only in MEDI-559 recipients; shedding occurred in 56%subjects, primarily post dose 1. A functional immune response was observed in 59% and 9% MEDI-559 and placebo recipients, respectively, by an RSV microneutralization assay. Vaccine take, assessed by proportion that shed vaccine-type virus or had a seroresponse against RSV, was seen in 95% MEDI-559 subjects. MEDI-559 is therefore biologically active and immunogenic in this seronegative pediatric population. Although the frequency of SSs and AEs was not considered clinically significant, the increase in medically attended lower respiratory illnesses in the vaccine group warrants expanded safety studies.

**Trial Registration:**

ClinicalTrials.gov NCT00767416

## Introduction

Respiratory syncytial virus (RSV) is the leading cause of severe lower respiratory infection and hospitalization in infants and young children [1,2]. In the United States alone, an estimated 2.1 million children younger than 5 years of age seek treatment for RSV infection annually [3]. In a 4-year observational study of predominantly healthy children (5 years or younger), acute respiratory infections associated with RSV accounted for 20% of hospitalizations, as well as 18% of emergency department visits and 15% of office visits [3]. The primary treatment for RSV infection is supportive care, and efforts to prevent the infection focus only on prophylaxis for high-risk infants. Therefore, a vaccine for RSV is urgently needed.

Attempts at developing an RSV vaccine have been unsuccessful. Children who received a formalin-inactivated RSV vaccine in the 1960s experienced enhanced RSV disease when subsequently infected with wild-type RSV [4]. The ideal immune response to a RSV vaccine should closely mimic the natural immune response to wild-type infection [5], and may be achieved by using a live attenuated vaccine approach. Earlier attempts in developing a live attenuated RSV vaccine identified cold-passaged (cp) and temperature-sensitive (ts) mutations and deletions (Δ) of nonessential genes that, when combined, resulted in attenuated vaccine candidates [6]. Vaccine development has been halted for several RSV vaccine candidates because of over- or underattenuation of the vaccine virus [6,7]. A precursor of MEDI-559, *cpts*248/404, was immunogenic, but caused nasal congestion that interfered with feeding in young infants [7]. The challenge remains to find the right balance of attenuation, safety, and immunogenicity.

This study followed the population and dosage level used in previous clinical studies conducted with rA2*cp*248/404/1030ΔSH, which is closely related to MEDI-559 [8]. MEDI-559 differs from the previously tested rA2cp248/404/1030ΔSH by 39 silent (non-coding) nucleotide substitutions dispersed throughout the virus genomes, including a substitution of the TTA codon at *ts* marker 248 with the CTG codon. As previously described, these viruses have comparable *in vitro* and *in vivo* phenotypes [9]. MEDI-559 is being developed under a Cooperative Research and Development Agreement by MedImmune, and the National Institute of Allergy and Infectious Diseases (National Institutes of Health).

## Methods

### Ethics Statement

Written informed consent was obtained from each parent or legal guardian before study entry or conduct of any protocol-specific activity. This study was conducted in accordance with the principles of the Declaration of Helsinki, the International Conference on Harmonisation Guideline for Good Clinical Practice, applicable laws and requirements, and conditions required by United States Food and Drug Administration. This study was approved by the appropriate Institutional Review Boards. Names and addresses of the Institutional Review Boards are listed in the Supporting Information (see Sites and IRBs S1). 

### Study Design

The protocol for this trial and supporting CONSORT checklist are available as supporting information; see Checklist S1 and Protocol S1. This was a randomized (1:1), double-blind, placebo-controlled phase 1 study performed at 28 sites in the United States (ClinicalTrials.gov identifier, NCT00767416). Randomization was managed centrally through an interactive voice response system. The study was initially designed with 2 cohorts: cohort 1 enrolled healthy subjects 5 to <24 months of age who were RSV-seronegative at baseline, and healthy unscreened subjects 1 to 3 months of age were intended for enrollment in cohort 2. Due to unavailability of clinical trial material, enrollment was closed after randomization of 116 of the planned 160 subjects into cohort 1, and cohort 2 was not enrolled. 

The primary objective of this study was to evaluate the tolerability and safety profile of 3 doses of MEDI-559 in healthy, RSV-seronegative children 5 to <24 months of age. Secondary objectives of this study assessed vaccine shedding, immunogenicity, genotypic and phenotypic stability, and long-term safety through at least one RSV season. 

### Study Population

Eligible participants were healthy full-term children 5 to <24 months of age who were RSV-seronegative at baseline, identified by a commercial enzyme-linked immunosorbent assay (ELISA) kit. Exclusion criteria included fever ≥38°C within 7 days prior to randomization; acute illness or nasal congestion; weight ≤5th percentile for age at day of randomization; use of immunosuppressive agents (including steroids); or receipt of any live virus or inactivated vaccine within 28 or 14 days before randomization, respectively. Additional restrictions included direct or indirect contact with infants <6 months of age, a pregnant caregiver, or immunocompromised individuals within 28 days after each dose.

### Study Vaccine

Eligible subjects received 3 doses of investigational product approximately 2 months apart. A 0.2-mL dose (0.1 mL per nostril) of 10^5±0.5^ fluorescent focus units (which is equivalent to approximately 10^5.3±0.5^ median tissue culture infective dose [TCID_50_] per mL) of MEDI-559 or placebo was administered as intranasal drops. Placebo was visually indistinguishable from MEDI-559, and both were stored frozen at or below -60°C.

### Safety

The primary safety evaluations included solicited symptoms, unsolicited adverse events (AEs), serious AEs (SAEs), and medically attended lower respiratory illnesses (MA-LRIs) from administration of investigational product through 28 days after each dose. A MA-LRI was defined as a clinical diagnosis made by a healthcare provider which included ≥1 of the following: wheezing, bronchiolitis, bronchitis, croup, pneumonia, rales, rhonchi, and apnea. Solicited symptoms were collected daily on a diary card by the parent/caregiver and included the following: runny/stuffy nose, cough, drowsiness, fever (defined as ≥38°C), irritability/fussiness, loss of appetite/decreased urine output, laryngitis (oropharyngeal irritation), and epistaxis. SAEs, MA-LRIs, and significant new medical conditions (SNMCs) were collected through 365 days after randomization. All AEs, including MA-LRIs, were assessed by the site investigator for severity and relationship to study vaccination.

### Immunogenicity Assessment

Sera were collected at baseline and following vaccination to determine the levels of RSV-neutralizing antibodies and RSV F-specific immunoglobulin (Ig)G and IgA levels. Due to the limits in the quantity of serum that could be collected from the infants, not all subjects had RSV antibody levels determined at all time points. Prior to enrollment, RSV seronegative children were identified utilizing a semi-qualitative commercial ELISA kit (Immuno-Biological Laboratories, Minneapolis, MN) that used purified F and G proteins as antigen. The assay was performed according to the manufacturer’s instructions. Children whose results were in the “negative” range were considered seronegative for the purposes of study inclusion. The ELISA was selected as a screening assay because it is a high-throughput assay and the results could rapidly be made available to sites, thereby facilitating the scheduling of enrollment and vaccination. 

The primary immune response was evaluated by measuring functional antibody responses by a RSV microneutralization assay using green fluorescent protein expressing RSV-A2 to infect Vero cells in the absence of exogenous complement. Heat-inactivated serum samples were tested in 2-fold serial dilutions at a starting dilution of 1:5. Neutralizing antibody titers were reported as the reciprocal of the highest dilution of serum that, compared with non-neutralized virus suspensions added to cells, resulted in a 60% reduction in the virus particle counts in infected cells. The lower limit of detection was 1:5 and the dynamic range of the assay was 1:5 to 1:5,120. In addition, serum was collected 28 days post dose 1 and post dose 2 in a small subset of subjects to assess the immune response after each of 3 doses, provided the subjects’ legal guardians consented to additional blood draws. Seroresponse was defined as a ≥4-fold rise from baseline in RSV-neutralizing antibody titer. Laboratory-confirmed wild-type RSV infection resulted in censoring those data from the immunologic assessment (4 subjects in vaccine group and 5 subjects in placebo group).

As an exploratory analysis, RSV F-specific serum IgG and IgA antibodies were measured at baseline and 28 days post dose 3 using the Meso Scale Discovery (MSD) platform (Meso Scale Diagnostics, Rockville, MD). RSV F-specific antibodies in serum samples were measured using purified recombinant RSV F lacking the transmembrane region and cytoplasmic tail [10] coated on the surface of an MSD plate. Subject serum samples were serially diluted in a 3-fold manner and transferred to the RSV F-coated MSD plates. Serum antibodies bound to RSV F were then detected using either a goat anti-human IgG or IgA antibody conjugated ruthenium tag (SULFO-TAG^TM^). Relative light units emitted from the ruthenium tagged detection antibody are proportional to the amount of IgG or IgA bound to RSV F. The F-specific IgG or IgA antibody titer was calculated for each serum sample using a simple linear regression using log_10_ transformed relative luminescence units and log_3_ transformed sample dilutions. F-specific IgG and IgA endpoint titers were calculated for each serum sample using the simple linear regression analysis, and the assay-specific cutoff defined as the signal 8-fold or 4-fold higher than the mean background signal for IgG and IgA respectively. Final antibody titers were reported as the log_3_ antibody titer raised to the power of 3 and multiplied by the sample dilution (sample titer = (dilution factor)*3^log_3_ titer). Seroresponse was defined as a ≥4-fold rise from baseline in RSV F-specific antibody titer. 

### Vaccine-Type Virus Detection

Nasal wash samples were collected to assess vaccine virus replication in the upper respiratory tract. Samples were collected on days 7 (range, 7–10), 12 (12–18), and 28 (28–34) after each dose. Additional nasal wash specimens were collected at unscheduled illness visits for subjects with respiratory symptoms occurring within 28 days of dosing and for all MA-LRIs that occurred within 365 days after dose 1. The collected samples were stabilized with an equal volume of M4 viral transport media (Thermo Scientific, Lenexa, KS), snap-frozen, and stored at -70°C or below until shipment to MedImmune.

Nucleic acid was extracted from nasal wash specimens and tested for the presence of RSV A and B using real-time quantitative reverse transcriptase polymerase chain reaction (qRT-PCR; Text S1 and Tables S1 and S2). The RSV A positive samples were then tested to determine if the sample was wild-type RSV A or MEDI-559 using a RT-PCR assay that determined the presence or absence of the SH gene (wt RSV/MEDI-559 ΔSH assay; Text S2 and Tables S3 and S4). When MEDI-559 virus was identified, the *ts* phenotype of the shed vaccine virus was evaluated, and the viral titer quantified by TCID_50_. For shed vaccine virus with an evaluable *ts* phenotype, virus was sequenced at the known *ts* loci to probe for genetic changes. 

For MA-LRI events, viral nucleic acid was isolated from stabilized nasal wash samples, and the detection of wild-type respiratory viruses was performed using qRT-PCR assays that detect influenza virus type A and B; RSV type A and B; parainfluenza viruses 1, 2, and 3; and human metapneumovirus (Respiratory Virus Detection Assays; Text S3 and Table S5). In parallel, the nucleic acid was analyzed for the presence or absence of the MEDI-559 vaccine virus by the ΔSH assay.

### Temperature Sensitivity/Phenotype Characterization

To determine the temperature sensitivity phenotype of shed MEDI-559, samples confirmed to contain MEDI-559 by the ΔSH assay and containing no wild-type RSV subtype A or B virus, were assessed for the ability to form plaques at 32°C, 35°C, and 39°C using an anti-RSV F fluorescence-staining plaque assay. *Ts* samples were defined as samples that showed ≥100-fold plaque reduction between 32°C and 35°C, *ts* intermediate (*tsi*) samples were defined as samples that showed a <100-fold plaque reduction between 32°C and 35°C, and samples defined as not *ts* phenotype grew at 32°C, 35°C, and 39°C.

### Genotypic Stability

The genotypic stability of the *ts* loci at 248, 404, and 1030 was assessed by Sanger sequencing to determine the nucleotide sequences [11,12]. PCR DNA was amplified from the shed vaccine viral nucleic acid followed by sequencing using primers specific to each locus. The sequences at the *ts* markers from the shed vaccine virus were compared with those of the administered vaccine.

### Median Tissue Culture Infective Dose Assay

The TCID_50_ assay, a Vero cell-based infectivity assay, was employed to determine the viral titers of shed MEDI-559 vaccine virus. Serial 10-fold dilutions of nasal wash specimens were applied to 96-well plates containing confluent monolayers of Vero cells, incubated for 7 days at 32°C, and immunostained with RSV-specific antibodies. Titers of shed virus were calculated using a modified Karber formula based on the number of wells containing RSV-infected cells as described by Reed and Muench [13]. 

### Statistical Analysis

Analyses were descriptive in nature, and no formal statistical comparisons were made.

## Results

### Study Subjects

**Figure 1:**
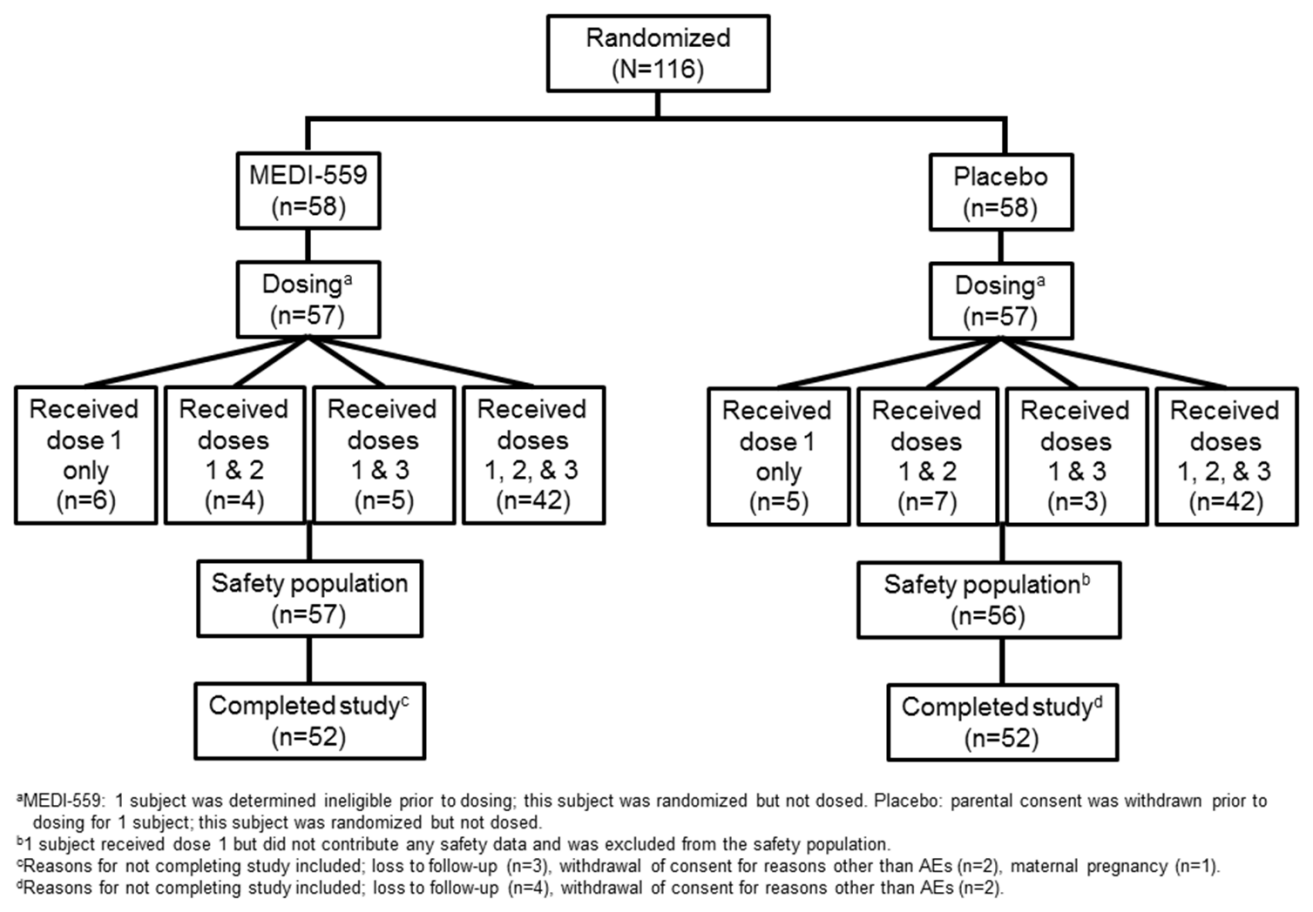
Subject disposition (CONSORT flowchart).

In total, 231 subjects were enrolled and screened for RSV; of these, 147 (64%) were seronegative for RSV using a RSV F and G antigen-specific ELISA. From this RSV-seronegative cohort, 116 subjects met all eligibility criteria and were randomized into cohort 1 from October 24, 2008, to December 13, 2010, at 28 sites throughout the United States (Figure 1). Out of 116 subjects, 114 received 1 dose and 72% of subjects received all 3 doses in each arm. Overall, 104 of 116 (90%) subjects were followed up for 365 days after randomization. 

Baseline demographic characteristics were similar between the MEDI-559 and placebo groups (Table 1). The median age was 7 and 8 months in the placebo and MEDI-559 group respectively; most subjects were female and white.

**Table 1:** Demographics of study subjects.

	**MEDI-559 (n=58)**	**Placebo (n=58)**
Mean (SD) age, mo	9.4 (4.5)	9.2 (4.1)
Median age, mo	8.0	7.0
Male, n (%)	24 (41.4)	25 (43.1)
Hispanic or Latino ethnicity, n (%)	18 (31.0)	24 (41.4)
Race, n (%)		
White	35 (60.3)	35 (60.3)
Black or African American	10 (17.2)	7 (12.1)
Other	13 (22.4)	16 (27.6)

### Safety

Solicited symptoms were common in both the MEDI-559 and placebo arms within 28 days of dosing. Most solicited symptoms occurred at similar frequencies between the 2 treatment groups; 48/57 (84%) of MEDI-559 subjects and 51/56 (91%) of placebo subjects experienced ≥1 solicited symptom overall (Table 2). The most commonly reported symptoms for all doses combined (overall) were runny/stuffy nose (MEDI-559: 72%; placebo: 88%), cough (MEDI-559: 56%; placebo: 52%), and irritability/fussiness (MEDI-559: 56%; placebo: 54%). No medically significant differences were observed between vaccine and placebo groups when examined by dose. Most (96%) solicited symptoms reported were mild in severity in both placebo and MEDI-559 arms. In the MEDI-559 arm, subjects who did not shed vaccine virus reported a higher frequency of solicited symptoms as compared to subjects who did shed vaccine virus (92% vs 78%).

**Table 2:** Solicited symptoms through day 28 after each dose.

	**Solicited Symptoms Through Day 28 After Each Dose, n (%)**							
	**Dose 1**		**Dose 2**		**Dose 3**		**Overall**	
**Solicited Symptom**	**MEDI-559 (n=57)**	**Placebo (n=56)**	**MEDI-559 (n=46)**	**Placebo (n=49)**	**MEDI-559 (n=47)**	**Placebo (n=45)**	**MEDI-559 (n=57)**	**Placebo (n=56)**
Total events, n	543	497	393	345	284	254	1220	1096
Subjects reporting ≥1 event (%)	42 (73.7)	37 (66.1)	31 (67.4)	34 (69.4)	26 (55.3)	30 (66.7)	48 (84.2)	51 (91.1)
Runny/stuffy nose (%)	35 (61.4)	29 (51.8)	25 (54.3)	28 (57.1)	21 (44.7)	24 (53.3)	41 (71.9)	49 (87.5)
Irritability/fussiness (%)	27 (47.4)	19 (33.9)	11 (23.9)	14 (28.6)	16 (34.0)	12 (26.7)	32 (56.1)	30 (53.6)
Cough (%)	18 (31.6)	20 (35.7)	17 (37.0)	12 (24.5)	12 (25.5)	7 (15.6)	32 (56.1)	29 (51.8)
Drowsiness (%)	11 (19.3)	14 (25.0)	4 (8.7)	8 (16.3)	5 (10.6)	7 (15.6)	16 (28.1)	21 (37.5)
Fever (%)								
38.0°C–38.5°C	8 (14.0)	7 (12.5)	6 (13.0)	5 (10.2)	5 (10.6)	4 (8.9)	18 (31.6)	15 (26.8)
38.6°C–39.5°C	3 (5.3)	4 (7.1)	4 (8.7)	6 (12.2)	0 (0.0)	2 (4.4)	7 (12.3)	10 (17.9)
39.6°C–40.5°C	1 (1.8)	2 (3.6)	1 (2.2)	0 (0.0)	0 (0.0)	0 (0.0)	2 (3.5)	2 (3.6)
>40.5°C	0 (0.0)	0 (0.0)	0 (0.0)	0 (0.0)	0 (0.0)	0 (0.0)	0 (0.0)	0 (0.0)

Unsolicited AEs were reported frequently in the 28 days after dosing and occurred in 38/57 (67%) of MEDI-559 recipients and 32/56 (57%) of placebo recipients. The most frequently reported AEs in the MEDI-559 and placebo arms were upper respiratory tract infection (URI) (MEDI-559: 35% vs placebo: 23%), otitis media (26% vs 18%), vomiting (18% vs 11%), diarrhea (14% vs 7%), and teething (11% vs 9%). All of these AEs were reported at slightly higher frequencies in the MEDI-559 arm compared with placebo. URI and otitis media were examined in more detail because the frequencies were higher in the MEDI-559 arm and could plausibly be linked to vaccine replication. There was no apparent association between vaccine virus shedding and otitis media or URI. Although a numeric imbalance was observed, the imbalances were not compelling when events were examined by dose (otitis media: dose 1, MEDI-559: 12% vs placebo: 9%; dose 2, 13% vs 12%; and dose 3, 9% vs 7%; URI: dose 1, MEDI-559: 18% vs placebo: 14%; dose 2, 17% vs 12%; and dose 3, 15% vs 9%). Most AEs were mild or moderate in severity (MEDI-559: 98%; placebo: 100%) and judged by the investigator to be unrelated to the investigational product.

Throughout the course of the study, 9 subjects in the MEDI-559 arm and 6 subjects in the placebo arm experienced MA-LRIs. Within 28 days after dosing, 5/57 (9%) MEDI-559 recipients experienced 6 MA-LRI events (wheezing, n=3; bronchiolitis, n=2; and croup, n=1) and among placebo recipients, 1/56 subject (2%) had wheezing (Table 3). None of the subjects with an MA-LRI had vaccine virus detected in nasal wash samples collected contemporaneously with the onset of the event. Wild-type RSV was detected in 3 MA-LRIs in the MEDI-559 arm, compared with 1 event in the placebo arm. After day 28 through the 365 days after randomization, an equal number of MA-LRI events occurred in both groups. Six MA-LRIs occurred in 5 subjects in the MEDI-559 group (bronchiolitis, n=3 [4 events in 3 subjects]; wheezing, n=2) and 5 subjects in the placebo group also had 6 MA-LRIs (bronchiolitis, n=2; wheezing, n=2; bronchitis, n=1; croup, n=1). Of note, there was no evidence of enhanced RSV disease (classically described as severe disease upon exposure to wild-type RSV leading to hospitalization and death).

**Table 3:** Medically attended lower respiratory illness among MEDI-559 and placebo recipients.

	**MA-LRI Term**	**Dose**	**Days Post Dosing**	**Virology**
**≤28 days post any dose**				
MEDI-559 (n=6)	Wheezing^a^	1	12	No virus detected
	Wheezing	1	10	RSV B
	Wheezing	1	28	No virus detected
	Bronchiolitis^a^	2	2	RSV A/Influenza A
	Bronchiolitis	2	10	No virus detected
	Croup	3	2	RSV A
Placebo (n=1)	Wheezing	3	14	RSV B
**>28 days post any dose**				
MEDI-559 (n=6)	Bronchiolitis^b^	1	29	No virus detected
	Wheezing^a^	1	54	RSV A/Influenza A
	Bronchiolitis^b^	3	36	Influenza B
	Bronchiolitis	3	75	No sample collected
	Bronchiolitis	3	102	Influenza B
	Wheezing	3	126	No virus detected
Placebo (n=6)	Wheezing^c^	1	30	RSV B/Influenza B
	Wheezing	1	55	No virus detected
	Bronchitis^c^	3	35	HMPV
	Croup	3	59	No virus detected
	Bronchiolitis	3	77	No sample collected
	Bronchiolitis	3	153	No sample collected^d^

HMPV, human metapneumovirus; MA-LRI, medically attended lower respiratory illness; NW, nasal wash; RSV, respiratory syncytial virus

a Subject had 3 MA-LRI events

b Subject had 2 MA-LRI events

c Subject had 2 MA-LRI events

d RSV positive by outside clinical lab

SAEs were balanced between the 2 groups. Two subjects in the vaccine arm had SAEs: 1 subject had Bell’s palsy, and another subject was hospitalized for bronchiolitis. The subject with Bell’s palsy developed a left facial droop 210 days post dose 2 that resolved within 3 weeks without complications. Because of the temporal latency and the occurrence of a precedent acute viral illness, the event was assessed as not related to vaccination. The subject hospitalized with bronchiolitis was found to have mild wheezing at the time of dose 2; however, the dose was administered. This subject developed bronchiolitis 2 days post dose 2, which was judged to be an SAE possibly related to the investigational product and led to permanent discontinuation. A nasal wash collected at the time of the MA-LRI SAE was positive for wild-type RSV A and influenza A. This subject also had 2 MA-LRIs of wheezing prior to the bronchiolitis SAE. The first event occurred 12 days after dose 1 and the second event started on the day dose 2 was administered (54 days after dose 1). Three subjects in the placebo arm had SAEs: altered mental status, accidental ingestion, and acute gastroenteritis with hypokalemia in 1 subject.

Two SNMCs occurred in MEDI-559 recipients (chronic cough, remotely related to investigational product; and reactive airway disease, not related to investigational product). Three SNMCs occurred among placebo recipients (peanut allergy, asthma, and allergic rhinitis; none related to investigational product).

### Vaccine Virus Shedding

**Figure 2:**
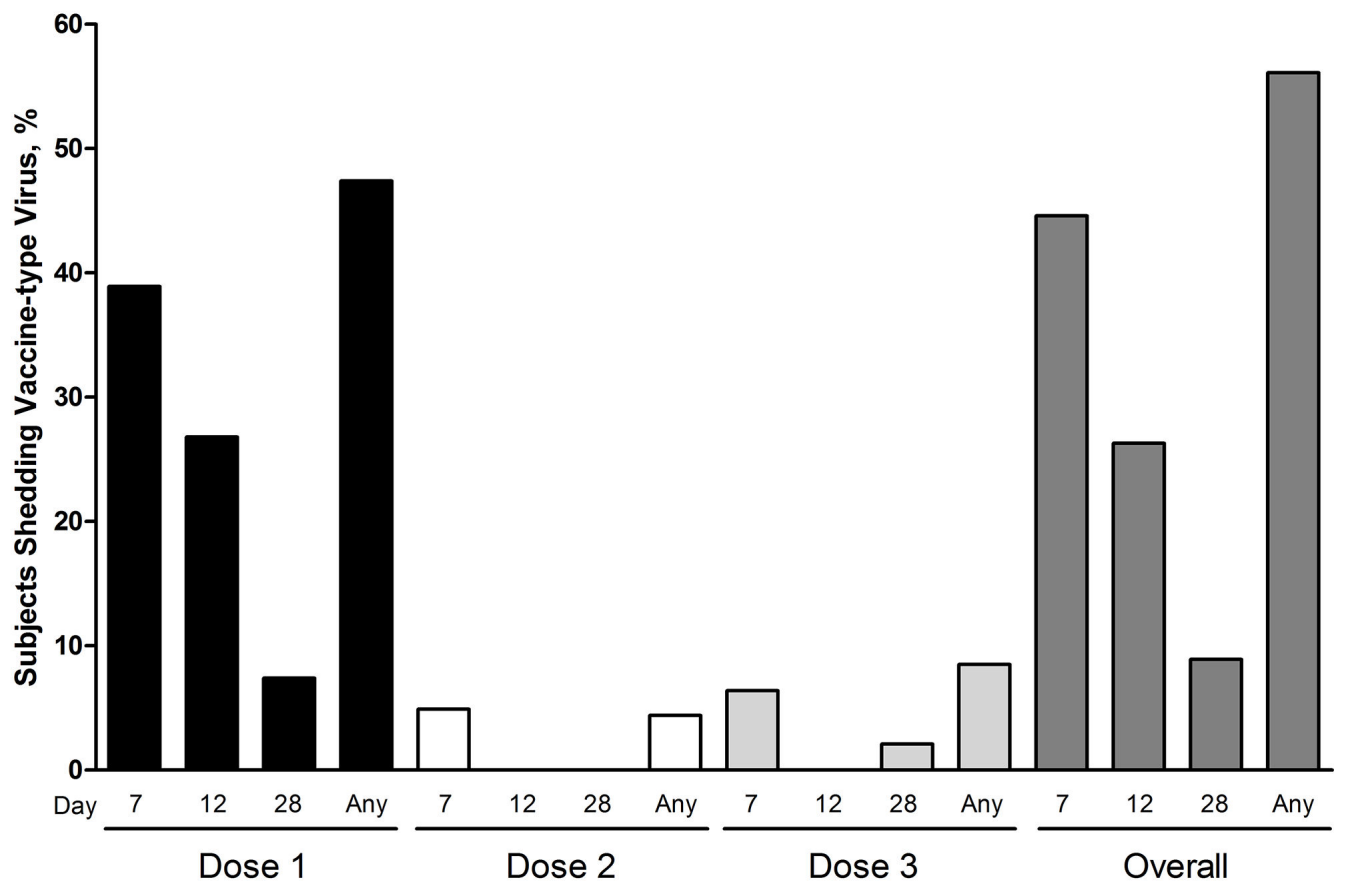
Proportion of MEDI-559 recipients who shed vaccine-type virus, stratified by study visit.

Vaccine-type virus was detected only in MEDI-559 recipients. Shedding of vaccine virus as detected by PCR occurred in 32/57 (56%) subjects, primarily after the first dose. The majority of shedding took place at days 7 and 12 post dose 1 (Figure 2). Shedding after doses 2 and 3 was limited; of the 5 subjects that shed after dose 2 or 3, only 1 had shed previously. Recovered viral titers were low; the mean viral titer was 1.3 log_*10*_ TCID_*50*_ /mL (range, 0.9–3.1). Peak viral titers were detected in 2 subjects after dose 1: one after day 7 and one after day 12. Viral titers for the few subjects who shed after dose 2 or 3 were generally lower than for subjects that shed after dose 1. Of the 56 subjects in the shedding population, only 46 subjects had baseline microneutralization results; 3 were excluded due to insufficient sample collection at baseline, and 7 were excluded because they did not have a post-vaccination sample collected within the pre-specified window. When stratified by baseline microneutralization antibody titer, 15/27 (56%) of the MEDI-559 subjects with a baseline titer of ≤10 shed vaccine-type virus, compared with 11/19 (58%) for those with a baseline antibody titer of >10. 

### Temperature-Sensitivity Phenotype and Genotype Stability

Forty-seven nasal wash samples from 32 subjects that shed vaccine virus were tested in a temperature sensitivity phenotyping assay. Thirty-five of the 47 samples were not evaluable because of low virus titer, so only 12 nasal wash samples generated temperature sensitivity phenotype results. Seven samples were identified as *ts* growing at 32°C only, and 5 were identified as *tsi* growing at 32°C and 35°C. None of the samples were non-*ts* or wild-type phenotype, as none grew at 39°C.

The 12 nasal wash samples with temperature sensitivity phenotype results were sequenced at *ts* loci 248, 404, and 1030. Eight of the 12 samples had the expected sequences at the *ts* loci (ie, no sequence changes were observed). Four samples from 4 subjects had sequence changes, and each of these samples had a *tsi* phenotype. One sample had a substitution containing mixtures of Leu/Ser at the 248 locus, 1 sample had a Tyr substitution at 1030 locus, and 2 samples had changes at both the 404 (M2-1 gene start) and 1030 loci (Asn Tyr). A fifth sample with a *tsi* phenotype had no identifiable genotypic sequence changes. There were no MA-LRIs or AEs in proximity to when the *tsi* samples were collected.

### Immunogenicity

A seroresponse (≥4-fold rise in RSV-neutralizing antibody titer from baseline) post dose 3 was observed in 59% (24/41) of MEDI-559 recipients compared with 9% (3/35) of placebo recipients by microneutralization assay (Table 4). Geometric mean titers (GMTs; MEDI-559: 64.8; placebo: 6.9) and geometric mean fold-rises (GMFRs; MEDI-559: 7.4; placebo: 0.7) in neutralizing serum antibodies demonstrate that MEDI-559 was immunogenic compared with placebo (Table 4). The ability of MEDI-559 to induce an immune response was inversely correlated with the baseline anti-RSV antibody response. Because the subjects were screened for study eligibility using a relatively insensitive commercial ELISA, some of the enrolled subjects had a detectable baseline RSV microneutralization response. For subjects in the immunogenicity population with baseline microneutralization response ≤10 (n=23), the seroresponse was 83% (GMT: 88; GMFR: 21), whereas for subjects with baseline microneutralization responses >10 (n=18), the seroresponse was 28% (GMT, 44; GMFR, 2).

**Table 4:** GMTs, GMFRs, and seroresponse in neutralizing serum antibodies against RSV^**a**^.

	**MEDI-559 Vaccinees**				**Placebo Vaccinees**			
	**n**	**GMT^b^ (95% CI)**	**GMFR^c^ (95% CI)**	**4-Fold Rise^d^, % (95% CI**)	**n**	**GMT^b^ (95% CI)**	**GMFR^c^ (95% CI)**	**4-Fold Rise^d^, % (95% CI**)
Microneutralization assay								
Baseline	46	8.3 (6.3, 11.2)			42	9.4 (6.8, 13.5)		
Post dose 1	9	27.2 (8.2, 93.3)	4.0 (0.9, 20.7)	37.5 (8.5, 75.5)	13	5.6 (4.0, 7.9)	0.8 (0.7, 0.9)	0.0 (0.0, 24.7)
Post dose 2	8	99.4 (38.3, 257.7)	15.3 (3.0, 64.0)	75.0 (34.9, 96.8)	11	5.3 (3.6, 8.8)	0.7 (0.5, 1.1)	0.0 (0.0, 28.5)
Post dose 3	41	64.8 (40.7, 104.8)	7.4 (3.9, 13.7)	58.5 (42.1, 73.7)	35	6.9 (4.5, 11.7)	0.7 (0.4, 1.2)	8.6 (1.8, 23.1)
Anti-F IgA								
Baseline	46	23 (17, 31)			41	28 (18, 48)		
Post dose 3	41^e^	672.0 (411, 1,083)	28 (17, 45)	90.2 (77, 97)	35^f^	57 (29, 115)	2 (1, 3)	11 (3, 27)
Anti-F IgG								
Baseline	45	110(56, 222)			41	434(237, 776)		
Post dose 3	40	12,005(7,813, 17,596)	92(36, 230)	85(70, 94)	35	390(199, 794)	1(0, 2)	9(2, 23.)

CI, confidence interval; GMFR, geometric mean fold-rise; GMT, geometric mean titer; Ig, immunoglobulin; LOQ, limit of quantitation; RSV, respiratory syncytial virus.

a Serum samples collected after a confirmed wild-type RSV infection were excluded from the analysis. CIs were constructed by percentile-based bootstrap method. Samples were collected 28 days after the indicated dose.

b Serum RSV antibody titers lower than the LOQ (LOQ=5) were imputed as half of the LOQ.

c GMFR was the ratio of day 28 antibody to the baseline antibody.

d Clopper-Pearson exact.

e 4 subjects excluded for confirmed wild-type RSV infection and 1 for not having post-dose 3 samples available

f 5 subjects excluded for confirmed wild-type RSV infection and 2 for not having post-dose 3 samples available

Additional analyses of the immune response were examined using RSV F-specific serum IgG and serum IgA assays if serologic samples were available. Both assays demonstrated humoral immune responses to the F antigen among MEDI-559 recipients after 3 vaccinations (Table 4). For F-specific IgG, 85% (34/40) and 9% (3/35) of MEDI-559 and placebo recipients, respectively, demonstrated a post-dose 3 seroresponse. For IgA, 90% (37/41) of MEDI-559 and 11% (4/35) of placebo recipients generated a 4-fold rise to RSV F (Table 4). Overall, 95% of subjects developed a 4-fold rise to at least one of the measures of humoral immunity (F-specific IgG, IgA, or RSV microneutralization).

## Discussion

MEDI-559 is a live attenuated intranasal vaccine and therefore requires appropriate attenuation to ensure safety while maintaining sufficient replication to induce a protective immune response. In this study, vaccine take, as measured by seroresponse by any serology assay (IgG, IgA, RSV microneutralization), was 95%, which is important when attempting to immunize a population. Although the RSV microneutralization response was lower in this study compared with the results from the study by Karron et al. using a similar construct (rA2*cp*248/404/1030ΔSH) [8], the RSV F Igg and IgA responses were comparable (both >85%). Direct comparison of immunologic results is complicated because of methodological differences. A key difference between the 2 studies was the assay used to measure RSV-neutralizing antibodies. Karron et al. included exogenous complement in their assay, whereas the RSV-neutralizing antibody assay used in the present study did not use complement. It has previously been demonstrated that adding complement to an RSV-neutralizing antibody assay can increase antibody titers by as much as 4-fold [14], and caution should be taken comparing antibody GMTs between the 2 studies.

The magnitude of the microneutralization response (seroresponse, GMT, and GMFR) observed in this study was inversely correlated with the baseline antibody titers. A possible explanation may be that the attenuated vaccine virus was neutralized by the pre-existing antibodies and was therefore less capable of inducing a vigorous immune response. However, this is not consistent with the observation that a similar number of subjects shed vaccine-type virus irrespective of baseline microneutralization titer. Furthermore, the microneutralization seroresponse rate was similar in subjects who shed and those who did not (62% of subjects that shed vs 53% of those who did not). These inconsistencies may be explained by the heterogeneity of the vaccine virus recovery methods or by chance alone.

The shedding rate in this study (56%) was lower than previously reported [8]. Potential causes for low vaccine virus recovery include a sampling scheme that did not collect nasal wash specimens until day 7 after dosing and/or inconsistencies in sample collection and processing that could have affected the ability to detect vaccine virus. Our study was conducted at 28 sites compared with a single site used in the Karron et al. study [8]. Of interest, in both studies, the first dose appeared to immunize against replication of subsequent doses, as there was very little shed virus detected after the second or third doses. 

As previously reported, genetic changes associated with a *tsi* phenotype were observed in approximately 40% of recovered samples. Because of the high mutation rate of RNA viruses, this is not unexpected. One sample had a *tsi* phenotype but had no detectable changes in the known *ts* loci. It is possible that compensatory changes elsewhere in the genome led to this to phenotypic reversion, as has been described with other RNA viruses [9,15], or that the TSI virus was present in the sample below the limit of detection of the sequencing assay, but was detected in tissue culture after 7 days. Importantly, none of the vaccine viruses reverted to the wild-type phenotype, and none of the *tsi* revertants were temporally associated with MA-LRIs.

Solicited symptoms, AEs, and SAEs were generally balanced between the vaccine and placebo recipients and were not considered clinically significant in most cases. Importantly, these solicited symptoms were generally balanced after dose 1, when the majority of the vaccine replication was detected. The overall rate of stuffy/runny nose was comparable with the placebo group and was not clinically significant; however, this would need to be evaluated in a younger age cohort of obligate nose breathers. There was an increased rate of MA-LRIs occurring within 28 days after dosing in the MEDI-559 group compared with the placebo group. The very low number of MA-LRI events in the placebo arm (2%) within 28 days of dosing is significantly lower than what has been observed in other similar pediatric RSV vaccine trials [16] and unpublished data (ClinicalTrials.gov identifier NCT00686075), in which the rate of MA-LRI events in placebo recipients ranged from 10% to 18%. Because no vaccine virus was recovered contemporaneously with the MA-LRIs, the biological mechanism is not readily apparent. A similar observation has been reported for a different mucosal live attenuated vaccine. Belshe et al. reported that live-attenuated influenza vaccine administered to 6- to 11-month-old children resulted in an increase in LRI within 42 days of dosing [17]. The relatively low number of subjects in this study does not allow for a determination of whether the excess MA-LRI cases occurred by chance or were a result of vaccination, as was observed following vaccination with live-attenuated influenza vaccine [17].

The current study has certain limitations that should be considered. Although the size of the study is appropriate for this age group given the early stage of product development, it was not sufficient to fully characterize the safety profile or determine with certainty if imbalances between treatment groups occurred by chance alone. Another limitation, intrinsic to the study design, is that the immune response was exclusively based on antibody responses. The serology assays only measured the RSV-specific serum antibody response; however, a live attenuated vaccine is expected to induce a humoral, mucosal, and cellular response to the entire RSV antigenic repertoire. Finally, the study was conducted throughout the calendar year, so wild-type RSV circulated during the evaluation period that may have impacted the immunologic assessment. Serologic data collected from children who had PCR-confirmed wild-type RSV infection were removed from the immunogenicity dataset. However, not all the RSV infections were captured through the clinical testing paradigm, as evidenced by 9% of the placebo group mounting a 4-fold microneutralization response even after PCR-confirmed RSV subjects were censured (Table 4).

In conclusion, this study demonstrated that MEDI-559 is biologically active and immunogenic in this pediatric population. Solicited symptoms and AEs observed in this study were consistent with those seen in children of this age and were not clinically significant. However, the imbalance in MA-LRIs among MEDI-559 recipients compared with placebo recipients will require an expanded safety study to further explore this observation.

## Supporting Information

Checklist S1 **CONSORT Checklist.**
(DOCX)

Protocol S1 **Trial Protocol.**
(PDF)

Sites and IRBs S1 (DOCX)

Study Investigators S1 (DOCX)

Table S1 **Sequence of the primers and probes for RSV A and B detection.**
(DOCX)

Table S2 **RT-PCR conditions.**
(DOCX)

Table S3 **Wild-type RSV/MEDI-559 ΔSH assay primer sequences.**
(DOCX)

Table S4 **Wild-type RSV/MEDI-559 ΔSH assay RT-PCR condition.**
(DOCX)

Table S5 **Sequence of the primers and probes for respiratory virus detection.**
(DOCX)

Text S1 **RSV qRT-PCR.**
(DOCX)

Text S2 **Wild-type RSV/MEDI-559 ΔSH assay.**
(DOCX)

Text S3 **Respiratory virus detection assays.**
(DOCX)
