# Supplementary material for: Safety and Immunogenicity of a Live Attenuated RSV Vaccine in Healthy RSV-Seronegative Children 5 to 24 Months of Age
Source: PLoS One. 2013 Oct 29;8(10):e77104. doi: 10.1371/journal.pone.0077104 (PMC3812203; doi:10.1371/journal.pone.0077104)
Supplement: Protocol S1 — Trial Protocol. (PDF) [file pone.0077104.s002.pdf]

A Phase 1/2a, Randomized, Double-Blind, Placebo-Controlled Study to Evaluate the Safety, Tolerability, Immunogenicity, and Viral Shedding of MEDI-559, a Live Attenuated Intranasal Vaccine Against Respiratory Syncytial Virus in Healthy 1 to <24 Month-Old Children

Study Agent: MEDI-559

MedImmune Protocol Number: MI-CP147

IND Number: BB-IND 13786

Manufacturer: MedImmune
[redacted]
Phone: [redacted]
Fax: [redacted]

Sponsor: MedImmune

Medical Monitor: [redacted]
Associate Director, Clinical Research
MedImmune
Phone: [redacted]
Fax: [redacted]

Study Monitor: [redacted]

Principal Investigator Agreement:

I, the undersigned, have reviewed this protocol and I agree to conduct this protocol in accordance with Good Clinical Practice, the ethical principles set forth in the Declaration of Helsinki and with the U.S. Code of Federal Regulations governing the protection of human subjects (21 CFR 50), Institutional Review Boards (21 CFR 56) and the obligations of clinical investigators (21 CFR 312).

Signature___________________________________ Date_______________

Printed Name________________________________

LIST OF ABBREVIATIONS

AE	Adverse event
CFR	Code of Federal Regulations
cp	Cold-passaged
CRF	Case report form
CI	Confidence interval
CTM	Clinical Trial Material
CRADA	Cooperative Research and Development Agreement
EDC	Electronic data capture
F	Fusion glycoprotein
FDA	US Food and Drug Administration
HIPAA	Health Insurance Portability and Accountability Act
HIV	Human immunodeficiency virus
ICH	International Conference on Harmonisation
IEC	Independent Ethics Committee
IND	Investigational New Drug Application
IRB	Institutional Review Board
IRE	Immediate reportable event
MA-LRI	Medically attended lower respiratory illness
ml	Milliliter
mM	Millimolar
NIH	National Institutes of Health
pfu	Plaque-forming units
PID	Participant identification number
PIV	Parainfluenza virus
PIV3	Parainfluenza virus type 3
RSV	Respiratory syncytial virus
SAE	Serious adverse event
SMC	Safety Monitoring Committee
SNMC	Significant new medical condition
ts	Temperature-sensitive

LIST OF DEFINITIONS

MEDI-559	rA2cp248/404/1030ΔSH developed under a CRADA between MedImmune and the NIH
rA2cp248/404/1030ΔSH (NIH/Wyeth)	rA2cp248/404/1030ΔSH developed under a CRADA between Wyeth and the NIH

TABLE OF CONTENTS

STUDY ABSTRACT 7

1 INTRODUCTION 14
1.1 Background 14
1.2 Description of MEDI-559 14
1.3 Preclinical Experience with MEDI-559 15
1.4 Clinical Experience with rA2cp248/404/1030ΔSH (NIH/Wyeth) 15
1.5 Rationale for Study 17

2 STUDY OBJECTIVES AND OVERVIEW 18
2.1 Primary Objective 18
2.2 Secondary Objectives 19
2.3 Overview 19

3 STUDY PROCEDURES 20
3.1 Participant Selection 20
3.1.1 Inclusion Criteria 20
3.1.2 Exclusion Criteria 21
3.2 Randomization 23
3.2.1 Subject Randomization Procedures and Treatment Allocation 23
3.2.2 Blinding 23
3.3 Study Vaccine (MEDI-559 and Placebo) 24
3.3.1 Study Vaccine Supplies and Accountability 24
3.3.2 Treatment Regimens 25
3.3.3 Study Vaccine Dispensing 25
3.3.4 Administration of Study Vaccine 26
3.3.5 Concomitant Medications/Immunizations 26
3.4 Schedule of Subject Evaluations 28
3.5 Subject Evaluation Methods 34
3.5.1 Safety Evaluations 34
3.5.2 Viral Shedding Evaluation 35
3.5.3 Immune Response Evaluations 36
3.6 Completion of Study and Loss to Follow-up 36

4 SAFETY ASSESSMENT 37
4.1 Adverse Events 37
4.1.1 Definition of Adverse Events 37
4.1.2 Definition of Solicited Symptoms 37
4.1.3 Definition of Medically Attended Lower Respiratory Illness 38
4.1.4 Definition of Significant New Medical Conditions 38
4.1.5 Study Reporting Period for Solicited symptoms, AEs, MA-LRIs, and SNMCs 39
4.1.6 Study Reporting Period for Enhanced Disease Surveillance 39
4.1.7 Recording of Solicited symptoms, AEs, MA-LRIs and SNMCs 40

4.2 Serious Adverse Events ... 40
4.2.1 Definition of Serious Adverse Events ... 40
4.2.2 Study Reporting Period for Serious Adverse Events ... 41
4.2.3 Notification of Sponsor of Serious Adverse Events ... 41
4.2.4 Notification of Institutional Review Board or Independent Ethics Committee of Serious Adverse Events ... 42
4.2.5 Recording of Serious Adverse Events ... 42
4.3 Assessment of Severity ... 43
4.4 Assessment of Relationship ... 43
4.5 Other Events Requiring Immediate Reporting ... 44
4.6 Safety Management During the Study ... 44
4.7 Interruption or Discontinuation of Study Dosing in Individual Subjects ... 46
4.8 Interruption or Discontinuation of Overall Study Dosing and Randomization ... 48
4.9 Cohort Progression ... 49
4.10 Monitoring of Dose Administration ... 49

5 STATISTICAL CONSIDERATIONS ... 50
5.1 General Considerations ... 50
5.2 Sample Size ... 50
5.3 Subject Populations ... 51
5.4 Primary Endpoints ... 52
5.5 Secondary Endpoints ... 52
5.6 Other Analyses ... 52

6 DATA HANDLING AND MONITORING ... 53

7 HUMAN SUBJECTS ... 53
7.1 Ethics and Regulatory Considerations ... 53
7.2 Institutional Review Board (IRB) or Independent Ethics Committee (IEC) ... 54
7.3 Informed Consent ... 55

8 STUDY COMPLETION ... 56

9 PUBLICATIONS ... 56

10 CHANGES IN THE PROTOCOL ... 56

11 REFERENCES ... 57

APPENDIX A Severity Grading Table for Solicited Symptoms and Adverse Events ... 58

APPENDIX B Respiratory Distress Severity Grading Table ... 60

APPENDIX C Events for Which a Nasal Wash Specimen is Required ... 61

APPENDIX D WHO Simplified Weight-Per-Age Field Tables 62

APPENDIX E Summary of Changes to the Protocol 64

STUDY ABSTRACT

TITLE:

A Phase 1/2a, Randomized, Double-Blind, Placebo-Controlled Study to Evaluate the Safety, Tolerability, Immunogenicity, and Viral Shedding of MEDI-559, a Live Attenuated Intranasal Vaccine Against Respiratory Syncytial Virus in Healthy 1 to <24 Month-Old Children

RATIONALE:

Respiratory syncytial virus (RSV) is the most important cause of viral lower respiratory tract illness in infants and children worldwide (Hall 2001). Palivizumab (Synagis®), a commercially available monoclonal antibody against the RSV F protein, prevents serious RSV disease in premature and other high-risk infants but is not indicated in healthy children. Since RSV infects and can cause disease in otherwise healthy infants, development of vaccines for the prevention of RSV disease is a public health priority (Murphy 2002).

A rA2cp248/404/1030ΔSH vaccine construct developed by the National Institutes of Health (NIH) was evaluated by Wyeth Pharmaceuticals in adults and children, including infants, under separate IND held by Wyeth. This construct will be referred to here as rA2cp248/404/1030ΔSH (NIH/Wyeth). MEDI-559, a closely related rA2cp248/404/1030ΔSH vaccine manufactured by MedImmune, is being co-developed by MedImmune and NIH under a Cooperative Research and Development Agreement (CRADA). MEDI-559 differs from the previously tested rA2cp248/404/1030ΔSH (NIH/Wyeth) vaccine by 39 silent base pair substitutions that do not cause any alterations of predicted amino acid sequence and result in no discernable phenotypic differences.

Phase 1 clinical studies were conducted by Wyeth to evaluate the safety and immunogenicity of rA2cp248/404/1030ΔSH (NIH/Wyeth) in seropositive human subjects (Karron 2005). In adults, the vaccine was safe and well tolerated but was non-immunogenic and failed to replicate. In RSV-seropositive children, rA2cp248/404/1030ΔSH (NIH/Wyeth) was well tolerated and highly restricted in replication. After vaccination, ≥4-fold increases in antibody titers occurred rarely (15% in the highest dose group), suggesting that this vaccine is minimally infectious and minimally immunogenic in RSV experienced populations.

The safety of the vaccine was further confirmed in randomized, double-blind, placebo-controlled trials in seronegative children (Karron 2005). A total of 21 seronegative children 6-24 months of age received one dose of either $10^{4.3}$ pfu or $10^{5.3}$ pfu of rA2cp248/404/1030ΔSH (NIH/Wyeth). The vaccine was safe and well tolerated. Viral shedding was monitored throughout the trial in

seronegative children, and 8/8 subjects (100%) who received the higher dose ($10^{5.3}$ pfu) shed virus compared to 8/13 (62%) of those who received $10^{4.3}$ pfu. In summary, a dose of $10^{5.3}$ pfu appeared to be more infectious and immunogenic than a dose of $10^{4.3}$ pfu in this population without evidence of increased reactogenicity.

A two dose trial of rA2cp248/404/1030ΔSH (NIH/Wyeth) was performed to evaluate the safety, tolerability, viral shedding profile and immunogenicity of the vaccine candidate in young infants who are the target population for MEDI-559. A total of 32 infants 4-12 weeks of age were enrolled to receive two doses of either $10^{4.3}$ or $10^{5.3}$ pfu 4-8 weeks apart. The vaccine was safe and well tolerated. Shedding of vaccine virus appeared to be dose dependent with the first vaccination priming for reduced shedding after second vaccination.

These clinical data support the further development of the MEDI-559 construct as a potential RSV vaccine. The epidemiology of RSV disease, with a disproportionate burden of disease borne by infants in the first year of life, suggests that the target age group for vaccination should be very young infants (Lee 2005).

The overall objective of the clinical development program is to evaluate the safety and efficacy of MEDI-559 for the prevention of serious RSV disease in infants and young children. The trials will evaluate the MEDI-559 vaccine using an equivalent dose in the same populations that were previously studied by Wyeth. The proposed expanded Phase 1/2a study will evaluate the safety, tolerability, immunogenicity and viral shedding of three doses of MEDI-559, first in 5 to <24-month old seronegative children and then in healthy young infants not screened for baseline serostatus. This trial will confirm the clinical safety and viral shedding profile of the new clinical trial material and generate expanded safety information on this vaccine to support a subsequent Phase 2b proof-of-concept efficacy trial.

OBJECTIVES:

Primary Objective

The primary objective of this study is to describe the 28-day post final dose safety and tolerability of three doses of MEDI-559 at 10^5 FFU when administered to healthy RSV seronegative children 5 to <24 months of age and to healthy infants 1 to <3 months of age regardless of baseline serostatus.

Secondary Objectives

The secondary objectives of this study are:

1. To describe the incidence and magnitude of MEDI-559 shedding after each dose
2. To evaluate the immune response generated by multiple doses of MEDI-559

3. To evaluate the genotypic and phenotypic stability of recovered vaccine virus
4. To describe the incidence of serious RSV disease in vaccinated subjects through 365 days after randomization
5. To describe the safety of MEDI-559 through 365 days post Dose 1

DESIGN:

This is a randomized, double-blind, placebo-controlled, multi-dose, Phase 1 /2a multi-center trial to evaluate the safety, tolerability, viral shedding, immunogenicity, and genotypic and phenotypic stability of MEDI-559 in RSV seronegative infants 5 to <24 months of age and in infants 1 to <3 months of age regardless of baseline serostatus. MEDI-559 will be administered at a dose of 10^5 FFU on a 0, 2, 4 month schedule to two cohorts of subjects in a step-wise fashion (see Section 4.9 for cohort progression scheme). The target sample size for this study is 320 subjects, with 160 subjects 5 to <24 months of age enrolled into Cohort 1 and 160 subjects 1 to <3 months of age enrolled into Cohort 2. This design follows the population and dose level used in previous clinical trials conducted with rA2cp248/404/1030ΔSH (NIH/Wyeth) (Karron 2005).

Each cohort will be randomized 1:1 (MEDI-559 to placebo) and stratified by site. Cohort 1 will initiate dosing at 10^5 FFU MEDI-559. The Medical Monitor and Safety Monitoring Committee (SMC) will review blinded safety data from the first 40 subjects enrolled in Cohort 1 for the 28 days following administration of the first dose of vaccine. Safety data for review includes solicited symptoms, adverse events (AEs), serious adverse events (SAEs), and medically attended lower respiratory illness (MA-LRIs). The SMC may also choose to review data by A/B grouping (treatment A vs. treatment B) or by unblinded grouping (MEDI-559 vs. placebo). If no safety concerns are noted, Cohort 2 will initiate dosing at 10^5 FFU MEDI-559.

Enrollment into Cohort 2 will be halted after approximately 40 subjects have been randomized. The Medical Monitor and SMC will review available blinded safety data through 28 days post Dose 1 for these 40 subjects. If no safety concerns are noted, the remainder of Cohort 2 will be enrolled.

Subjects will be followed for 365 days after randomization. This will standardize the follow-up period for all subjects across all sites and locations globally, regardless of local RSV epidemiology, and it ensures that all subjects are followed through a full RSV season after dosing.

SUBJECT POPULATION:

Inclusion Criteria

Subjects must meet *all* of the following criteria:

1. Male or female whose age on the day of randomization falls within one of the two age cohorts:
   Cohort 1: 5 to <24 months (reached their 5th month birthday but not yet reached their 2nd year birthday);
   Cohort 2: 1 to <3 months (>28 days of age and not yet reached their 3rd month birthday)
2. Cohort 1 only: Subject is seronegative to RSV at screening as determined by ELISA; or the legal representative is willing to provide access to data documenting that the subject was screened for another MedImmune trial after written informed consent was obtained, and that the subject is seronegative to RSV within 21 days prior to randomization into MI-CP147 as determined by ELISA at MedImmune
3. Subject was the product of normal full term pregnancy (defined as 36-42 weeks gestation)
4. Subject is in general good health
5. Subject's legal representative is available by telephone throughout the trial period
6. Written informed consent and HIPAA authorization (if applicable) obtained from the subject's legal representative
7. Subject's legal representative is able to understand and comply with the requirements of the protocol as judged by the investigator
8. Subject is available to complete the follow-up period, which will be 365 days after randomization.
9. Subject's legal representative is willing and able to bring the subject to the study site for evaluation of respiratory illness in accordance with the protocol

Exclusion Criteria

Subjects must have *none* of the following:

1. Any fever (≥100.4°F [≥38.0°C]), regardless of route within 7 days prior to randomization
2. Acute illness (defined as the presence of moderate or severe signs and symptoms) at the time of randomization
3. Moderate or severe nasal congestion that in the investigator's opinion could prevent intranasal delivery of vaccine
4. Cohort 1 only: weight ≤5th percentile for age on the day of randomization (see APPENDIX D)
5. Cohort 2 only: history of low birth weight (ie, <2500 grams at birth) or weight ≤5th percentile for age on the day of randomization (see APPENDIX D)
6. Any drug therapy (chronic or other) within 7 days prior to randomization or expected receipt through 28 days after each study vaccine dosing, except that infrequent use of over-the-

counter medications for the symptomatic treatment of common childhood illnesses (eg, pain relievers, decongestants, or cough suppressants) are permitted according to the judgment of the investigator

7. Any current or expected receipt of immunosuppressive agents including steroids (≥2 mg/kg per day of prednisone or its equivalent, or ≥20 mg/day if the subject weighs >10 kg, given daily or on alternate days for ≥14 days); children in this category should not receive study vaccine until immunosuppressive agents including corticosteroid therapy have been discontinued for ≥30 days; the use of topical steroids is permitted according to the judgment of the investigator
8. History of receipt of blood transfusion or expected receipt through 28 days after final study vaccine dosing
9. History of receipt of immunoglobulin products or expected receipt through 28 days after final study vaccine dosing
10. Receipt of any investigational drug within 60 days prior to randomization or expected receipt through 28 days after final study vaccine dosing
11. Receipt of any live virus vaccine (excluding rotavirus vaccine) within 28 days prior to randomization or expected receipt within a 28-day window around any study vaccine dose
12. Receipt of any inactivated (eg, non-live) vaccine or rotavirus vaccine within 14 days prior to randomization or expected receipt within a 14-day window around any study vaccine dose
13. Known or suspected immunodeficiency, including HIV infection
14. Living in the same home or enrolled in the same classroom at full-time day care with infants <6 months of age within 28 days after each dose (only one child per household may be enrolled into the study)
15. Contact with pregnant caregiver within 28 days after each dose
16. Living in a household with someone who is immunocompromised within 28 days after each dose; the subject should also avoid close contact with immunocompromised individuals for 28 days after each dose
17. Living in a household with someone who works in the healthcare field and who has direct patient care responsibilities within 28 days after each dose
18. Living in a household with someone who is a day care provider or preschool teacher for children <6 months of age within 28 days after each dose
19. History of allergic reaction to any component of the study vaccine
20. Previous medical history or evidence of an intercurrent or chronic illness that, in the opinion of the investigator, may compromise the safety of the subject
21. Known or suspected active or chronic hepatitis infection
22. History of medical diagnosis of asthma, reactive airway disease, wheezing requiring medication, bronchoconstriction or treatment with a β_2 agonist (eg, albuterol), cystic fibrosis,

chronic lung disease of prematurity (eg, bronchopulmonary dysplasia), chronic pulmonary disease, medically confirmed apnea, hospitalization for respiratory illness, or mechanical ventilation for respiratory illness (excludes elective mechanical ventilation during surgery for subjects in Cohort 1)

23. Immediate family member or household member who is an employee of the clinical study site or who is otherwise involved with the conduct of the study

24. Any condition that, in the opinion of the investigator, might interfere with study vaccine evaluation

Note: An individual who initially is excluded from study participation based on one or more of the above time-limited criteria (eg, acute illness) may be reconsidered for enrollment once the condition has resolved contingent on the subject continuing to meet all other entry criteria.

TREATMENT:

Cohort 1 (5 to <24 months):	N=80	MEDI-559 at 10^5 FFU at 0, 2, and 4 months
	N=80	Placebo at 0, 2, and 4 months
Cohort 2 (1 to <3 months):	N=80	MEDI-559 at 10^5 FFU at 0, 2, and 4 months
	N=80	Placebo at 0, 2, and 4 months

SUBJECT EVALUATION AND FOLLOW-UP:

Safety will be evaluated by the collection of solicited symptoms, adverse events (AEs), serious adverse events (SAEs), concomitant medication use, medically attended lower respiratory illness (MA-LRI, defined in Section 4.1.3) and significant new medical conditions (SNMCs, defined in Section 4.1.4). The subject's legal representative will complete a worksheet to bring to study clinic visits to facilitate collection of potential solicited symptoms, AEs, SAEs, and concomitant medications and vaccines. AEs, and SAEs will be assessed for severity and relationship to study vaccine. Solicited symptoms will be assessed for severity and SNMCs will be assessed for relationship to study vaccine.

A nasal wash specimen will be collected at Days 7, 12, and 28 following each dose to assess vaccine virus replication and vaccine-like viral shedding. Additional unscheduled nasal wash specimens will be collected from subjects who require an unscheduled illness visit in accordance with protocol Section 3.4. All subjects who experience an MA-LRI during the entire 365 study period will be asked to return to the clinical site for a nasal wash specimen collection. Nasal wash specimens will be assayed for the presence of vaccine-like virus and for the presence of certain respiratory pathogens. All vaccine virus that is recovered from nasal wash samples will

be quantified to evaluate the level of replication as a marker of attenuation. Vaccine virus that is isolated will also be evaluated for genotypic stability.

A serum sample will be collected at the Study Day 0 visit for the baseline assessment of immune response. Subjects whose legal representative have provided written informed consent for the collection of additional samples will have 3 additional serum samples collected at the Day 7 and Day 28 visits after Dose 1 and at the Day 28 visit after Dose 2 to assess immune response. A serum sample will be obtained for all enrolled subjects 28-34 days after the final dose to assess immune response.

ASSESSMENT OF ENDPOINTS:

The primary endpoints of safety and tolerability of MEDI-559 will be measured by:

- Incidence of solicited symptoms from administration of study vaccine through 28 days following each dose
- Incidence of adverse events from administration of study vaccine through 28 days following each dose
- Incidence of MA-LRIs from administration of study vaccine through 28 days following each dose
- Incidence of SAEs from administration of study vaccine through 28 days post final dose

The secondary endpoints include the following:

- Incidence and magnitude of MEDI-559 shedding at Days 7, 12, and 28 after each dose
- Percentage of subjects with a seroresponse to RSV after the final dose (defined as a ≥4-fold rise from baseline in neutralizing antibody titer, regardless of baseline serostatus)
- Description of the genotypic and phenotypic stability of recovered vaccine virus
- Description of the potential for immunologic enhancement of RSV disease by evaluation of MA-LRIs associated with RSV infection through 365 days after randomization
- Incidence and description of SAEs through 365 days after randomization
- Incidence and description of SNMCs through 365 days after randomization

1 INTRODUCTION

1.1 Background

Respiratory syncytial virus (RSV) is the most important cause of viral lower respiratory tract illness in infants and children worldwide (Hall 2001). Palivizumab (Synagis®), a commercially available monoclonal antibody against the RSV F protein, prevents serious RSV disease in premature and other high-risk infants but is not indicated in healthy children. Since RSV infects and can cause disease in otherwise healthy infants, development of vaccines for the prevention of RSV disease is a public health priority (Murphy 2002). Live attenuated RSV vaccines have been in development for several decades and represent a promising strategy for protecting young infants against serious RSV disease. Intranasal delivery mimics natural infection and offers several advantages, including the potential for the induction of both systemic and mucosal immunity (Durbin 2003). In addition, live viral vaccines have not been associated with the enhanced RSV disease that occurred in trials of formalin-inactivated RSV vaccine (Chin 1969). Finally, the peak of severe disease and mortality from RSV occurs in infants 2 to 6 months of age, and live intranasal vaccines have been shown to be immunogenic in this target population of young infants despite immunologic immaturity and the presence of maternal antibodies (Karron 2003; Karron 2005; Wright 2000).

1.2 Description of MEDI-559

MEDI-559 (rA2cp248/404/1030ΔSH) is being developed as a live, attenuated, intranasal vaccine for the prevention of lower respiratory tract disease caused by RSV in young infants. MEDI-559 was engineered using reverse genetics. It contains multiple attenuating genetic elements that are responsible for its temperature sensitivity (*ts*) and attenuation (*att*) properties: a set of 5 mis-sense mutations in the N, F and L proteins that attenuate *cp*[cold-passaged]RSV; the 248 and 1030 modifications that are separate mis-sense mutations in the L protein that lower the restrictive temperature and confer a *ts* phenotype and attenuate the virus; 404, a combination of a nucleotide substitution in the gene-start transcription signal of the M2 gene and a mis-sense mutation in L protein that together confer *ts*; and ΔSH, a deletion of the entire SH gene that confers *att*. The presence of multiple nucleotide substitutions and a gene deletion contribute to the phenotypic stability of the vaccine.

A rA2cp248/404/1030ΔSH vaccine construct developed by the National Institutes of Health (NIH) was evaluated by Wyeth Pharmaceuticals in adults and children, including infants, under separate IND held by Wyeth. This construct will be referred to here as rA2cp248/404/1030ΔSH (NIH/Wyeth). MEDI-559, a closely related rA2cp248/404/1030ΔSH vaccine manufactured by

MedImmune, is being co-developed by MedImmune and NIH in a Cooperative Research and Development Agreement (CRADA). MEDI-559 differs from the previously tested rA2cp248/404/1030ΔSH (NIH/Wyeth) vaccine by 39 silent base pair substitutions that do not cause any alterations of predicted amino acid sequence and result in no discernable phenotypic differences. The majority of these substitutions removed changes previously introduced during manufacture of the rA2cp248/404/1030ΔSH (NIH/Wyeth) vaccine construct.

1.3 Preclinical Experience with MEDI-559

MEDI-559 and rA2cp248/404/1030ΔSH (NIH/Wyeth) are identical in all preclinical characterization studies. MEDI-559 has been shown to be genetically stable after multiple passages in vitro, to possess the same *ts* phenotype in vitro as the NIH vaccine, to be equivalently attenuated in BALB/c mice and to lead to blunting of viral replication upon wild-type challenge. rA2cp248/404/1030ΔSH (NIH) and MEDI-559 also have comparable immunogenicity in BALB/c mice. Refer to the Investigator's Brochure for additional information.

1.4 Clinical Experience with rA2cp248/404/1030ΔSH (NIH/Wyeth)

Single Dose of rA2cp248/404/1030ΔSH (NIH/Wyeth) in Seropositive Adults and Children 15-59 Months of Age

Phase 1 clinical studies were conducted by Wyeth to evaluate the safety and immunogenicity of rA2cp248/404/1030ΔSH (NIH/Wyeth) in seropositive human subjects (Karron 2005). The vaccine was initially evaluated in adults (N=16) and seropositive children 15-59 months of age (N=25). All subjects received a single dose of $10^{4.3}$ or $10^{5.3}$ pfu of rA2cp248/404/1030ΔSH (NIH/Wyeth) vaccine. In adults, the vaccine was safe and well tolerated but was non-immunogenic and failed to replicate. In RSV-seropositive children, rA2cp248/404/1030ΔSH (NIH/Wyeth) was well tolerated and highly restricted in replication. One seropositive recipient of rA2cp248/404/1030ΔSH (NIH/Wyeth) experienced pneumonia on Study Day 5; this child did not have evidence of infection with vaccine virus, and enterovirus was isolated on Study Days 4 and 5. After vaccination, ≥4-fold increases in antibody titers occurred rarely (15% in the highest dose group), suggesting that this vaccine is minimally infectious and minimally immunogenic in RSV experienced populations.

Single Dose of rA2cp248/404/1030ΔSH (NIH/Wyeth) in Seronegative Children 6-24 Months of Age

The safety of the vaccine was further confirmed in randomized, double-blind, placebo-controlled trials in seronegative children (Karron 2005). A total of 21 seronegative children 6-24 months of

age received one dose of either $10^{4.3}$ pfu or $10^{5.3}$ pfu of rA2cp248/404/1030ΔSH (NIH/Wyeth). The vaccine was safe and well tolerated. Otitis media was observed more frequently in vaccine recipients (4/21 or 19%) than in placebo recipients (0/16), but it occurred sporadically throughout the study period, was not consistently associated with vaccine virus shedding, and occurred more frequently at the lower dose. Lower respiratory tract illnesses (LRTIs) were not observed in vaccine recipients. Overall, the frequency of reported adverse events was consistent with those expected for the pediatric population tested and with events observed in placebo recipients.

Viral shedding was monitored throughout the trial in seronegative children, and 8/8 subjects (100%) who received the higher dose ($10^{5.3}$ pfu) shed virus compared to 8/13 (62%) of those who received $10^{4.3}$ pfu. Immunologic response was also better in the higher dose group. In the $10^{4.3}$ pfu dose group, 12 children had evaluable data, and 4 children (33%) generated a serum antibody response to the vaccine while 5 children (42%) demonstrated a mucosal IgA response. In contrast, all 8 children (100%) who received the $10^{5.3}$ pfu dose demonstrated a serum antibody response to the vaccine with 88% developing neutralizing antibodies. The dose response in infectivity and immunogenicity suggests that increases in dose may be one strategy for enhancement of the immune response to highly attenuated RSV vaccines. In summary, a dose of $10^{5.3}$ pfu appeared to be more infectious and immunogenic than a dose of $10^{4.3}$ pfu in this population without evidence of increased reactogenicity.

Study in Infants 1 to 3 months of Age of a Less Attenuated RSV Candidate

In a previous study of a less attenuated RSV candidate vaccine, *cpts* 248/404, 17 out of 24 infants 1 to 3 months of age (71%) developed nasal congestion that occurred most typically from 8 to 12 days after vaccination and that precluded further development of the vaccine (Wright 2000). Symptoms of congestion were linked temporally with the peak of virus shedding with 15 out of 18 infants who shed $>10^3$ pfu/ml experiencing congestion, fussiness while trying to sleep, and mild difficulty with feeding which lasted approximately 24 hours.

Two Doses of rA2cp248/404/1030ΔSH (NIH/Wyeth) in Infants 4-12 Weeks of Age

A two dose trial of rA2cp248/404/1030ΔSH (NIH/Wyeth) was performed to evaluate the safety, tolerability, viral shedding profile, and immunogenicity of the vaccine candidate in young infants who are the target population for MEDI-559. A total of 32 infants 4-12 weeks of age were enrolled to receive two doses of either $10^{4.3}$ or $10^{5.3}$ pfu 4-8 weeks apart. Rates of clinically significant nasal congestion were lower with this more attenuated vaccine and did not lead to any obligatory mouth breathing. In the $10^{4.3}$ pfu dose group, 19% of infants had congestion that interfered with sleeping or feeding after the first dose of vaccine and 43% after the second dose;

placebo rates were 0% and 40%. Rates were similar in the $10^{5.3}$ pfu dose group: 44% after the initial vaccination and 25% after the second vaccination. Two LRTIs occurred in vaccine recipients: both received the $10^{4.3}$ pfu dose, neither shed vaccine virus and, in both cases, an alternate etiologic agent was isolated (rhinovirus and PIV3).

Shedding of vaccine virus appeared to be dose dependent, with the first vaccination priming for reduced shedding after second vaccination. In the $10^{4.3}$ pfu group, 14 of 16 children received two doses of vaccine. A total of 11 children (69%) shed vaccine: 63% percent shed after the first dose and 29% after the second. All 16 subjects who received two doses of $10^{5.3}$ pfu shed vaccine: 94% shed after the first dose and 44% after the second. Of the 25 infants in both dosing groups who shed vaccine virus after the first dose of vaccine, 18 (72%) shed virus that retained the *ts* phenotype, 2 (8%) shed virus that had shifted to a *ts* intermediate phenotype (*tsi* – vaccine viruses which no longer exhibit a 2 log or greater restriction at 35-36 °C) and 5 shed virus with titers too low to allow for phenotyping. Neither infant who shed *tsi* virus experienced a LRTI. As the *tsi* viruses retained all of their non-*ts* attenuating mutations and 2 of the 3 *ts* mutations, it is likely that they remained highly attenuated.

As was seen with virus infectivity and consistent with dosing in the older seronegative children, antibody responses increased substantially in infants receiving the higher dose of vaccine. Only 6% of infants who received two $10^{4.3}$ pfu doses showed any antibody response to the vaccine compared to 44% of infants who received two $10^{5.3}$ pfu doses. Neutralizing antibodies developed in none of the infants who received the lower dose but in 19% of those who received the $10^{5.3}$ pfu dose.

RSV-seronegative children and infants enrolled in these studies were followed for RSV disease during the winters after vaccination. Enhanced disease was not observed with subsequent wild-type RSV infection (Karron 2005). This is consistent with all previously tested live attenuated mucosal and live parenteral RSV vaccines (Karron 1997, Karron 2005, Wright 2000, Wright 2007).

1.5 Rationale for Study

These clinical data support the further development of the MEDI-559 construct as a potential RSV vaccine. The epidemiology of RSV disease, with a disproportionate burden of disease borne by infants in the first year of life, suggests that the target age group for vaccination should be very young infants (Lee 2005). Data from dosing with rA2cp248/404/1030ΔSH (NIH/Wyeth) vaccine suggest that it is appropriately attenuated for these young infants. In addition, the vaccine is able to induce immune responses despite immunological immaturity and the presence of maternal antibodies. Safety data support dosing at $10^{5.3}$ pfu, as does the improved infectivity

and immunogenicity of this higher dose in both young and older infants. Dosing of MEDI-559 in this protocol will be at 10^5 fluorescence focus units (FFU) per dose, which was found to be essentially equivalent to a dose of $10^{5.3}$ pfu when the plaque and fluorescence focus assays were compared (mean FFA potency result was 0.372 $\log_{10}$ lower than mean plaque potency).

The extent of shedding of rA2cp248/404/1030ΔSH (NIH/Wyeth) and the limited seroconversion occurring after a second dose of vaccine in the target population of very young infants suggests that multiple vaccine doses will be required for adequate protection against wild-type virus exposure. A schedule of three doses each separated by 2 months is selected based on the immunologic immaturity of the youngest infants that is reflected in poor vaccine immunogenicity in the study of rA2cp248/404/1030ΔSH (NIH/Wyeth) and in other trials of live, intranasal viral vaccines (Karron 2005, Karron 2003). Delivering the third dose at approximately 6 months of age allows for a diminution of the effects of maternal antibody and of immunologic immaturity on vaccine responsiveness. The improved antibody responses observed to date in the older infants suggests that ending dosing at 6 months might provide durable immunity at least throughout the first year of life. A 2-month interval was selected because a significant minority of children (44%) shed after a second dose was administered 4-8 weeks after the first even though 15 of 16 children had shed after the first dose. In addition, a 2-month interval conforms to the currently recommended pediatric vaccination schedule.

The overall objective of the clinical development program is to evaluate the safety and efficacy of MEDI-559 for the prevention of serious RSV disease in infants and young children. The trials will evaluate the MEDI-559 vaccine using an equivalent dose in the same populations that were previously studied by Wyeth. This proposed expanded Phase 1/2a study will evaluate the safety, tolerability, immunogenicity and viral shedding of three doses of MEDI-559, first in 5 to <24-month old seronegative children and then in healthy young infants 1 to <3 months of age not screened for baseline serostatus. This trial will confirm the clinical safety and viral shedding profile of the new clinical trial material and generate expanded safety information on this vaccine to support a subsequent Phase 2b proof-of-concept efficacy trial.

2 STUDY OBJECTIVES AND OVERVIEW

2.1 Primary Objective

The primary objective of this study is to describe the 28-day post final dose safety and tolerability of three doses of MEDI-559 at 10^5 FFU when administered to healthy RSV seronegative children 5 to <24 months of age and to healthy infants 1 to <3 months of age regardless of baseline serostatus.

2.2 Secondary Objectives

The secondary objectives of this study are:

1. To describe the incidence and magnitude of MEDI-559 shedding after each dose
2. To evaluate the immune response generated by multiple doses of MEDI-559
3. To evaluate the genotypic and phenotypic stability of recovered vaccine virus
4. To describe the incidence of serious RSV disease in vaccinated subjects through 365 days after randomization
5. To describe the safety of MEDI-559 through 365 days post Dose 1

2.3 Overview

This is a randomized, double-blind, placebo-controlled, multi-dose, Phase 1/2a multi-center trial to evaluate the safety, tolerability, viral shedding, immunogenicity, and genotypic and phenotypic stability of MEDI-559 in RSV seronegative infants 5 to <24 months of age and in infants 1 to <3 months of age regardless of baseline serostatus. MEDI-559 will be administered at a dose of 10^5 FFU on a 0, 2, and 4 month schedule to two cohorts of subjects in a step-wise fashion (see Section 4.9 for cohort progression scheme). The target sample size for this study is 320 subjects, with 160 subjects 5 to <24 months of age enrolled into Cohort 1 and 160 subjects 1 to <3 months of age enrolled into Cohort 2. This design follows the population and dose level used in previous clinical trials conducted with rA2cp248/404/1030ΔSH (NIH) (Karron 2005).

Each cohort will be randomized 1:1 (MEDI-559 to placebo) and stratified by site. Cohort 1 will initiate dosing at 10^5 FFU MEDI-559. The Medical Monitor and Safety Monitoring Committee (SMC) will review blinded safety data from the first 40 subjects enrolled in Cohort 1 for the 28 days following administration of the first dose of vaccine. Safety data for review includes solicited symptoms, adverse events (AEs), serious adverse events (SAEs), and medically attended lower respiratory illness (MA-LRIs). The SMC may also choose to review data by A/B grouping (treatment A vs. treatment B) or by unblinded grouping (MEDI-559 vs. placebo). If no safety concerns are noted, Cohort 2 will initiate dosing at 10^5 FFU MEDI-559. Enrollment into Cohort 2 will be halted after approximately 40 subjects have been randomized. The Medical Monitor and SMC will review available blinded safety data through 28 days post Dose 1 for these 40 subjects. If no safety concerns are noted, the remainder of Cohort 2 will be enrolled.

The study will be conducted globally in multiple sites with dosing throughout the year. All subjects will be followed through 365 days after randomization to ensure that each subject has been followed through an RSV season.

Study Flow Diagram

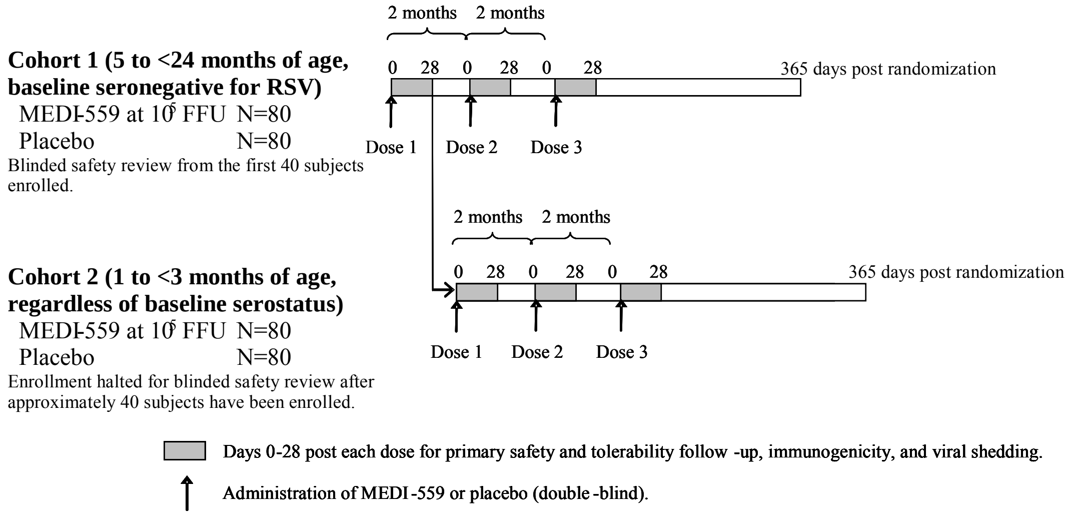

3 STUDY PROCEDURES

3.1 Participant Selection

The subjects in this study will be healthy male or female children 5 to <24 months of age who are baseline seronegative for RSV (Cohort 1) and healthy male or female children 1 to <3 months of age not screened for baseline serostatus (Cohort 2).

The legal representative of each subject will be counseled by the site investigator (physician) and/or study staff who will explain the study, address any questions and concerns, and secure written informed consent from the subject's legal representative for their child's participation in the study. Written informed consent will be obtained prior to conducting any screening or study procedures or administering study vaccine.

3.1.1 Inclusion Criteria

Subjects must meet *all* of the following criteria:

1. Male or female whose age on the day of randomization falls within one of the two age cohorts:
   Cohort 1: 5 to <24 months (reached their 5th month birthday but not yet reached their 2nd year birthday);
   Cohort 2: 1 to < 3 months (>28 days of age and not yet reached their 3rd month birthday)
2. Cohort 1 only: Subject is seronegative to RSV at screening as determined by ELISA; or the legal representative is willing to provide access to data documenting that the subject was screened for another MedImmune trial after written informed consent was obtained, and that the subject is seronegative to RSV within 21 days prior to randomization into MI-CP147 as determined by ELISA at MedImmune

3. Subject was the product of normal full term pregnancy (defined as 36-42 weeks gestation)
4. Subject is in general good health
5. Subject's legal representative is available by telephone throughout the trial period
6. Written informed consent and HIPAA authorization (if applicable) obtained from the subject's legal representative
7. Subject's legal representative is able to understand and comply with the requirements of the protocol as judged by the investigator
8. Subject is available to complete the follow-up period, which will be 365 days after randomization
9. Subject's legal representative is willing and able to bring the subject to the study site for evaluation of respiratory illness in accordance with the protocol

3.1.2 Exclusion Criteria

Subjects must have *none* of the following:

1. Any fever (≥100.4°F [≥38.0°C]), regardless of route within 7 days prior to randomization
2. Acute illness (defined as the presence of moderate or severe signs and symptoms) at the time of randomization
3. Moderate or severe nasal congestion that in the investigator's opinion could prevent intranasal delivery of vaccine
4. Cohort 1 only: weight ≤5th percentile for age on the day of randomization (see APPENDIX D)
5. Cohort 2 only: history of low birth weight (ie, <2500 grams at birth) or weight ≤5th percentile for age on the day of randomization (see APPENDIX D)
6. Any drug therapy (chronic or other) within 7 days prior to randomization or expected receipt through 28 days after each study vaccine dosing, except that infrequent use of over-the-counter medications for the symptomatic treatment of common childhood illnesses (eg, pain relievers, decongestants, or cough suppressants) are permitted according to the judgment of the investigator
7. Any current or expected receipt of immunosuppressive agents including steroids (≥2 mg/kg per day of prednisone or its equivalent, or ≥20 mg/day if the subject weighs >10 kg, given daily or on alternate days for ≥14 days); children in this category should not receive study vaccine until immunosuppressive agents including corticosteroid therapy have been discontinued for ≥30 days; the use of topical steroids is permitted according to the judgment of the investigator
8. History of receipt of blood transfusion or expected receipt through 28 days after final study vaccine dosing

9. History of receipt of immunoglobulin products or expected receipt through 28 days after final study vaccine dosing

10. Receipt of any investigational drug within 60 days prior to randomization or expected receipt through 28 days after final study vaccine dosing

11. Receipt of any live virus vaccine (excluding rotavirus vaccine) within 28 days prior to randomization or expected receipt within a 28-day window around any study vaccine dose

12. Receipt of any inactivated (eg, non-live) vaccine or rotavirus vaccine within 14 days prior to randomization or expected receipt within a 14-day window around any study vaccine dose

13. Known or suspected immunodeficiency, including HIV infection

14. Living in the same home or enrolled in the same classroom at full-time day care with infants <6 months of age within 28 days after each dose (only one child per household may be enrolled into the study)

15. Contact with pregnant caregiver within 28 days after each dose

16. Living in a household with someone who is immunocompromised within 28 days after each dose; the subject should also avoid close contact with immunocompromised individuals for 28 days after each dose

17. Living in a household with someone who works in the healthcare field and who has direct patient care responsibilities within 28 days after each dose

18. Living in a household with someone who is a day care provider or preschool teacher for children <6 months of age within 28 days after each dose

19. History of allergic reaction to any component of the study vaccine

20. Previous medical history or evidence of an intercurrent or chronic illness that, in the opinion of the investigator, may compromise the safety of the subject

21. Known or suspected active or chronic hepatitis infection

22. History of medical diagnosis of asthma, reactive airway disease, wheezing requiring medication, bronchoconstriction or treatment with a β_2 agonist (eg, albuterol), cystic fibrosis, chronic lung disease of prematurity (eg, bronchopulmonary dysplasia), chronic pulmonary disease, medically confirmed apnea, hospitalization for respiratory illness, or mechanical ventilation for respiratory illness (excludes elective mechanical ventilation during surgery for subjects in Cohort 1)

23. Immediate family member who is an employee of the clinical study site or who is otherwise involved with the conduct of the study

24. Any condition that, in the opinion of the investigator, might interfere with study vaccine evaluation

Note: An individual who initially is excluded from study participation based on one or more of the above time-limited criteria (eg, acute illness) may be reconsidered for enrollment once the condition has resolved contingent on the subject continuing to meet all other entry criteria.

3.2 Randomization

3.2.1 Subject Randomization Procedures and Treatment Allocation

Subjects will be screened by investigators to assess eligibility for entry into the study. A master log will be maintained of all screened subjects. When a subject arrives in the clinic for the Study Day 0 visit (day of initial study vaccination), the investigator will confirm that the subject meets all eligibility criteria. Subjects whose legal representative has signed an informed consent at screening and who meet eligibility criteria will be randomized into the study.

On the first day of dosing, subjects whose legal representative signed an informed consent and who meet all eligibility criteria (including re-assessment of all eligibility criteria on the day of dosing) will be randomized. For each cohort, subjects will be randomized at a 1:1 ratio (MEDI-559 to placebo) and randomization will be stratified by site. Subjects within each cohort for whom their legal representative has provided written informed consent for the collection of additional samples may have an additional three blood samples collected for immunogenicity evaluations. Randomization will be managed centrally through an interactive voice response system (IVRS).

A participant is considered to be randomized into the study upon assignment of a participant identification number (PID). All subjects entered into the study will be assigned a unique PID. Once assigned, the PID will be maintained by the same subject throughout the trial.

3.2.2 Blinding

This is a double-blind study. All protocol-associated MedImmune and Contract Research Organization (CRO) personnel including the medical monitor, biostatistician, project management, site monitors, and data managers will be blinded to treatment assignment with the exception of MedImmune staff responsible for laboratory assays. Staff responsible for laboratory assays have access to potentially unblinding information (such as seroconversion or shedding of vaccine virus) but will not have access to treatment randomization codes. These staff members must not reveal potentially unblinding information to anyone or participate in or be associated with the evaluation of study subjects.

In addition, the subject, the subject's legal representative, and the clinical site staff including the investigators, study nurses, and coordinators will be blinded. The subject's family member/legal representative will be permitted to be present at vaccine administration.

The vendor for packaging and labeling of the clinical supplies, IVRS personnel, MedImmune Clinical Research Pharmacy Service (CRPS) personnel, Unblinded Safety Physician and Safety Biostatistician (see Section 4.6), and designated persons in MedImmune Quality Assurance are the only individuals who will have potential access to a subject's treatment allocation. These individuals must not reveal randomization or treatment information to anyone or participate in or be associated with the evaluation of study subjects. In the event that the treatment allocation for a subject becomes known to the investigator or other study staff, the sponsor must be notified immediately by the investigator. For the purpose of unblinding due to any emergent safety issues, a representative from MedImmune Quality Assurance will perform an emergency unblinding according to MedImmune Standard Operating Procedure.

Both MEDI-559 and placebo will be supplied to the study center as identically packaged, single dose units in labeled syringes. The labels will not identify the treatment allocation or expiration date. MEDI-559 and placebo are identically labeled and indistinguishable in appearance.

Interim analyses by cohort may be performed after all subjects in a cohort reach 28 days post last scheduled dose. Selected MedImmune staff who are not directly involved with study conduct may be fully unblinded after completion of data collection through 28 days after the final dose in a cohort but prior to completion of collection of SAEs and SNMCs through 365 days post randomization to analyze data in preparation for the proof-of-concept trial. Additional unblinded interim safety analyses by cohort may be performed. For all analyses, MedImmune staff who are involved in the conduct of the study, site staff, subjects, and subjects' legal representatives will remain blinded to subject treatment assignments through completion of data collection through the entire 365 day follow-up period for each study cohort.

3.3 Study Vaccine (MEDI-559 and Placebo)

3.3.1 Study Vaccine Supplies and Accountability

The sponsor will provide the investigators with MEDI-559 and placebo. The filled syringes are frozen and stored -60°C or below. Study vaccine must be thawed prior to intranasal administration and should not be refrozen after thawing. Study vaccine will be delivered intranasally as droplets.

MEDI-559: MEDI-559 vaccine is supplied as a frozen preparation of live, attenuated rA2cp248/404/1030ΔSH virus filled into 0.5 ml Hypak®

SCF® (sterile, clean, fill) luer slip-tip syringes (used as a nasal dropper). Each 0.2 ml dose contains $10^{5\pm0.5}$ FFU (fluorescent focus units) of MEDI-559 in a sucrose phosphate glutamate buffer with cell culture media and residual host cell protein and DNA. MEDI-559 contains no preservatives.

Placebo Placebo is supplied as a frozen preparation filled into 0.5 ml Hypak® SCF® (sterile, clean, fill) luer slip-tip syringes (used as a nasal dropper). Each 0.2 ml dose contains sucrose phosphate buffer.

Accurate study vaccine accountability records must be maintained by site personnel. Upon completion of the study, all study vaccine accountability records will be returned to the sponsor. All unused study vaccine will be returned to MedImmune or disposed of upon authorization of MedImmune (refer to the CTM Manual for contact information and specific shipping instructions).

3.3.2 Treatment Regimens

Cohort 1 (5 to <24 months):	N=80	MEDI-559 at 10^5 FFU at 0, 2, and 4 months
	N=80	Placebo at 0, 2, and 4 months
Cohort 2 (1 to <3 months):	N=80	MEDI-559 at 10^5 FFU at 0, 2, and 4 months
	N=80	Placebo at 0, 2, and 4 months

3.3.3 Study Vaccine Dispensing

MEDI-559 and placebo will be supplied as identically packaged, single-dose units. Study vaccine units and outer packaging boxes will be labeled in accordance with current Good Manufacturing Practices guidelines. A multi-part label on the syringe will identify the protocol number and a unique study vaccine number. After the study vaccine is administered to the subject, one part of the label remains affixed to the syringe and the other part is removed and affixed to the subject's permanent study record retained at the study center.

In the event that an assigned syringe is broken or damaged, or if there is any concern about the integrity of the closed system of the syringe, a call will be made to the IVRS to provide the appropriate replacement syringe number when necessary. The replacement syringe will contain the same study material as the originally assigned syringe. Refer to the Clinical Trial Material (CTM) Manual for more detailed information regarding replacement procedures.

All supporting regulatory documentation as required by the Food and Drug Administration (FDA), other regulatory authorities, MedImmune, and other applicable local, legal, and

regulatory requirements must be in place and verified prior to shipment of study vaccine supplies to the clinical study center. Blinded study vaccine will be packaged on dry ice (-60°C) and upon request of MedImmune the distributor will ship study vaccine directly to the clinical study center by express courier. Receiving departments should be notified that rapid handling of the shipment is required. Upon receipt at the study site, study vaccine should be immediately transferred to a -60°C or below, non-frost-free freezer. It is the responsibility of the investigator to maintain daily temperature logs for the freezer (refer to the CTM Manual for details). The investigator will be provided with temperature monitors that record minimum/maximum temperatures, unless temperature monitors are already in place.

3.3.4 Administration of Study Vaccine

- Study vaccine must be thawed prior to administration. The vaccine may be thawed by holding the syringe in the palm of the hand and supporting the plunger rod with the thumb, or by exposing to room temperature for 5 minutes. The vaccine should be administered immediately upon thawing.
- A single administration comprises intranasal delivery of 0.2 ml total volume (0.1 ml into each nostril). While the subject is held in a semi-reclined or a horizontal position, half of the syringe contents will be administered into each nostril. The child should be held with the head tilted back for approximately one-half minute following dosing.
- Each syringe has a divider that allows delivery of half the contents of the syringe into one nostril. Removal of the divider allows delivery of the remaining volume into the other nostril.
- After administration, used study syringe must be placed immediately into locked containers or sealed bags.
- After vaccination all subjects will be observed for a minimum of 30 minutes by the study staff. Emergency management supplies (eg, AMBU bag, adrenaline [epinephrine], antihistamine) must be made available for the initial treatment of an allergic reaction if needed. Local reactions or systemic events must be recorded on the case report form (CRF).

3.3.5 Concomitant Medications/Immunizations

The following concomitant medication and immunizations will be recorded using the data capture instrument provided:

- All concomitant medications/immunizations received by the subject from immediately after administration of study vaccine through 28 days after each dose.
- All concomitant medications administered to treat any MA-LRI or SAE that occurs immediately after administration of study vaccine through 365 days after randomization.

- All doses of any rotavirus vaccine received by the subject from 28 days prior to the first study dose through 28 days after the final study dose.

The following concomitant medications are prohibited through the post dosing periods as indicated:

1. Any drug therapy (chronic or other) through 28 days after each study vaccine dosing, except that infrequent use of over-the-counter medications such as pain relievers are permitted. Subjects may receive medications to treat AEs as deemed necessary by the investigator or the subject's physician.

2. Any immunosuppressive agents including steroids (≥2 mg/kg per day of prednisone or its equivalent, or ≥20 mg/day if the subject weighs >10 kg, given daily or on alternate days for ≥14 days); children in this category should not receive study vaccine until immunosuppressive agents including corticosteroid therapy have been discontinued for ≥30 days; the use of topical steroids is permitted according to the judgment of the investigator

3. Blood transfusion through the final protocol-specified blood collection at 28 days after final study vaccine dosing

4. Immunoglobulin products through the final protocol-specified blood collection at 28 days after final study vaccine dosing

5. Any investigational drug through the final protocol-specified blood collection at 28 days after final study vaccine dosing

6. Any live virus vaccine (excluding rotavirus vaccine) within a 28-day window around any dose

7. Any inactivated (eg, non-live) vaccine or rotavirus vaccine within a 14-day window around any dose

Study subjects should receive all routine immunizations (including rotavirus) in accordance with the current recommendations of the pertinent national authority. A study subject will not forego or delay the receipt of any mandatory vaccine beyond the currently recommended immunization schedule according to local guidance (eg, in the USA, the recommendation of the Centers for Disease Control and Prevention, and the equivalent in other countries) due to study participation.

Infrequent use of over-the-counter medications, such as pain relievers, is permitted, but the use of prophylactic medications (eg, antipyretics/analgesics administered to minimize any potential

vaccine-related adverse event from the day of each study vaccine dosing through 28 days after each dose) should be discouraged.

The sponsor must be notified if any subject receives a prohibited concomitant medication.

3.4 Schedule of Subject Evaluations

Subjects who are assigned a PID will be followed according to the protocol, regardless of the number of doses of study vaccine received, through 365 days post randomization, unless consent for follow-up is withdrawn. The sponsor must be notified of all deviations from protocol visits or evaluations and these evaluations, if applicable, must be rescheduled or performed at the nearest possible time to the original schedule.

A schedule of screening and on-study visit procedures is presented in Table 1, followed by a detailed description of each visit. Subjects who have any of the following events will be brought in for an unscheduled illness visit for assessment and the collection of a nasal wash specimen for vaccine virus isolation, and, as appropriate, quantitation, genotyping, and phenotyping for attenuation reversion and for the identification of infections caused by non-vaccine respiratory viruses. Legal representatives will be instructed to arrange for unscheduled “illness” visits within 24 hours or as soon as possible after recognition of the event or a medically attended respiratory illness visit. Full definitions of the terms below are provided in APPENDIX C.

Symptoms within 28 days after any dose requiring unscheduled illness visit for nasal wash collection (see APPENDIX C for full definition of terms):

- Fever ≥100.4°F (≥38.0°C) regardless of route
- Cough for ≥2 consecutive days
- Runny nose/nasal congestion for ≥2 consecutive days
- Difficulty breathing (increased respiratory sounds, retractions, tachypnea, color change, grunting, nasal flaring, gasping)
- Medically attended visit for any acute respiratory illness including acute otitis media (AOM) or lower respiratory tract illness (MA-LRI) (as described in Section 4.1.3)

Events at any time during study participation (Day 0 through 365 days post randomization) requiring unscheduled illness visit for nasal wash collection:

- Any respiratory SAE (an SAE that is respiratory in nature according to the judgment of the investigator)
- Any related SAE (an SAE assessed as possibly, probably, or definitely related to study vaccine)
- Any MA-LRI (as described in Section 4.1.3, to monitor for enhanced disease during the entire 365 study period)

Table 1 Schedule of Subject Evaluations

	Study Day														
	Screen	0	7	12	28	56	7	12	28	56	7	12	28	365[h]	Unscheduled Illness Visit
		Dose 1	Post Dose 1			Dose 2	Post Dose 2			Dose 3	Post Dose 3				
Informed consent & HIPAA	X														
Verify eligibility	X	X[a,b]				X[a,b]				X[a,b]					
Medical history	X	X[a,c]				X[a]				X[a]					
Physical exam	X	X[a,c]	X[d]	X[d]	X[d]	X[a,d]	X[d]	X[d]	X[d]	X[a,d]	X[d]	X[d]	X[d]		X[d]
Current medication use	X	X[a,c]													
Blood for baseline serostatus[j]	X														
Blood for immune response[l]		X[a]	(X,[k])		(X[k])				(X[k])				X		
Randomize/Assign PID		X[a]													
Study vaccine administration[e]		X				X				X					
Nasal wash specimen	X		X	X	X		X	X	X		X	X	X		X[f]
Solicited symptoms, AEs, & concomitant medication use[g]		←——→				←——→				←——→					X[i]
MA-LRIs, SAEs, SNMCs		←——————————————→													X

Scheduled clinic visits to occur at screening (2-21 days prior to Dose 1); on Day 0 (Dose 1 administration), 56 days (±8 days) post Dose 1 (Dose 2 administration), and 56 days (±8 days) post Dose 2 (Dose 3 administration); and at 7-10, 12-18, and 28-34 days after each dose (blood and/or nasal specimen collection). Telephone contacts will occur on a monthly basis after the subject has completed the final scheduled clinic visits to assess the occurrence of MA-LRIs, SAEs, and SNMCs. A final telephone contact is scheduled for 365-379 days post final dose.

a. Perform prior to dosing.
b. Verify initial eligibility (Dose 1, Sections 3.1.1 and 3.1.2) and continuing eligibility (Dose 2 and Dose 3, Sections 4.7and 4.8).
c. Update screening medical history, physical exam (including body weight), and current medication use (any new findings since screening).
d. Targeted physical exam (temperature, examination of ears, nose, throat, regional lymph nodes, lung examination including respiratory rate, and other areas per medical history).
e. Intranasal administration of MEDI-559 or placebo, followed by a 30-minute observation for safety. Worksheets and thermometers will be distributed with instructions.
f. In addition to scheduled nasal wash specimens, samples for illness cultures will be collected within 24 hours or as soon as possible after illness onset or visit for medically attended respiratory illness, for Days 0-28 following each dose of study vaccine. Additional nasal specimens will be collected throughout the study for any MA-LRI. See Section 3.4 for details.
g. AEs and MA-LRIs that are ongoing at 28 days post dosing will be followed until resolution by weekly telephone contacts.
h. MA-LRIs, SAEs, and SNMCs will be collected from Day 0 through 365 days post randomization.
i. Information collected if the event requiring the illness visit occurs within 28 days post any dose, except as required for SAE report forms.
j. Only subjects in Cohort 1 (5 to <24 months of age) will be screened for baseline serostatus.
k. Only subjects whose legal representatives consent to additional blood draws for immunogenicity evaluation will have additional 3 blood draws for immunogenicity assessment; all other subjects will have 2 blood draws (Day 0 prior to Dose 1 and 28 days after Dose 3).

Screening

Note: All screening laboratory assessments must be performed within 2-21 days prior to randomization (2-21 days prior to Study Day 0). The screening evaluations may be carried out over more than one visit.

1. Obtain written informed consent and HIPAA (if applicable)
2. Verify eligibility
3. Perform screening medical history
4. Perform screening physical exam (including body weight)
5. Record current medication use
6. Collect blood for baseline serostatus: only subjects in Cohort 1 (5 to <24 months of age) will be screened for baseline serostatus
7. Collect nasal wash specimen

Study Day 0: Randomization and Study Vaccine Dose 1

Visit 1

1. Verify eligibility criteria (including seronegative status for RSV for Cohort 1)
2. Update medical history (any new findings since screening)
3. Update physical exam, including body weight (any new findings since screening)
4. Update current medication use (any new findings since screening)
5. Collect blood for baseline immune response (for all subjects in both cohorts)
6. Randomize and assign PID
   All of the above must be completed prior to study vaccine administration
7. Administer study vaccine and observe for at least 30 minutes
8. Record solicited symptoms, AEs, SAEs, MA-LRIs, SNMCs, and concomitant medication use
9. Inquire about MA-LRIs, SAEs, and SNMCs

Study Day 7: (7-10 days post dosing): Follow-up Post Dose 1

Visit 2

1. Perform targeted physical exam
2. Collect blood for immune response **for subjects participating in optional blood sampling**. **Written informed consent must have been obtained from each subject's legal representative prior to entry into the study for subjects scheduled to have additional blood draws performed for immunogenicity evaluation**.
3. Collect nasal wash specimen
4. Record solicited symptoms, AEs, and concomitant medication use
5. Inquire about MA-LRIs, SAEs, and SNMCs

Study Day 12: (12-18 days post dosing): Follow-up Post Dose 1

Visit 3

1. Perform targeted physical exam
2. Collect nasal wash specimen.
3. Record solicited symptoms, AEs, and concomitant medication use
4. Inquire about MA-LRIs, SAEs, and SNMCs

Study Day 28: (28-34 days post dosing): Follow-up Post Dose 1

Visit 4

1. Perform targeted physical exam
2. Collect blood for immune response **for subjects participating in optional blood sampling**. **Written informed consent must have been obtained from each subject's legal representative prior to entry into the study for subjects scheduled to have additional blood draws performed for immunogenicity evaluation**.
3. Collect nasal wash specimen
4. Record solicited symptoms, AEs, and concomitant medication use through 28 days post Dose 1
5. Inquire about MA-LRIs, SAEs, and SNMCs

56 Days Post Dose 1 (56 ± 8 days post dosing): Administration of Dose 2

Visit 5

1. Verify continuing eligibility criteria
2. Update medical history
3. Perform targeted physical exam
4. Update AEs and concomitant medication use that were continuing 28 days after Dose 1
   All of the above must be completed prior to study vaccine administration
5. Administer study vaccine and observe for at least 30 minutes
6. Record solicited symptoms, AEs, and concomitant medication use
7. Inquire about MA-LRIs, SAEs, and SNMCs

7 Days Post Dose 2 (7-10 days post dosing): Follow-up Post Dose 2

Visit 6

1. Perform targeted physical exam
2. Collect nasal wash specimen
3. Record solicited symptoms, AEs, and concomitant medication use
4. Inquire about MA-LRIs, SAEs, and SNMCs

12 Days Post Dose 2 (12-18 days post dosing): Follow-up Post Dose 2

Visit 7

1. Perform targeted physical exam
2. Collect nasal wash specimen
3. Record solicited symptoms, AEs, and concomitant medication use
4. Inquire about MA-LRIs, SAEs, and SNMCs

28 Days Post Dose 2 (28-34 days post dosing): Follow-up Post Dose 2

Visit 8

1. Perform targeted physical exam
2. Collect blood for immune response **for subjects participating in optional blood sampling**. **Written informed consent must have been obtained from each subject's legal representative prior to entry into the study for subjects scheduled to have additional blood draws performed for immunogenicity evaluation**.
3. Collect nasal wash specimen

4. Record solicited symptoms, AEs, and concomitant medication use through 28 days post dosing
5. Inquire about MA-LRIs, SAEs, and SNMC

56 Days Post Dose 2 (56 ± 8 days post dosing) Dose 2: Administration of Dose 3

Visit 9

1. Verify continuing eligibility
2. Update medical history
3. Perform targeted physical exam
4. Update AEs and concomitant medication use that were continuing 28 days after Dose 2
   All of the above must be completed prior to study vaccine administration
5. Administer study vaccine and observe for at least 30 minutes
6. Record solicited symptoms, AEs, and concomitant medication use
7. Inquire about MA-LRIs, SAEs, and SNMCs

7 Days Post Dose 3 (7-10 days post dosing): Follow-up Post Dose 3

Visit 10

1. Perform targeted physical exam
2. Collect nasal wash specimen
3. Record solicited symptoms, AEs, and concomitant medication use
4. Inquire about MA-LRIs, SAEs, and SNMCs

Day 12 (12-18 days post dosing): Follow-up Post Dose 3

Visit 11

1. Perform targeted physical exam
2. Collect nasal wash specimen
3. Record solicited symptoms, AEs, and concomitant medication use
4. Inquire about MA-LRIs, SAEs, and SNMCs

Day 28 (28-34 days post dosing): Follow-up Post Dose 3

Visit 12

1. Perform targeted physical exam
2. Collect blood for immune response (for all subjects in both cohorts)

3. Collect nasal wash specimen
4. Record solicited symptoms, AEs, and concomitant medication use through 28 days post dosing
5. Inquire about MA-LRIs, SAEs, and SNMCs

Once a Month (every 30 days ± 7 days) from Final Scheduled Visit through final scheduled telephone contact 12 months (365 days) after randomization

Telephone contacts

1. Inquire about SAEs, SNMCs, and MA-LRIs since previous visit or telephone contact (Note: final telephone contact must be scheduled for at least 365 days after randomization, and includes contacts with subjects who withdrew from dosing after randomization)

Unscheduled Illness Visit from 0- 28 days after a dose of study vaccine

Visit PRN

1. Perform targeted physical exam
2. Collect nasal wash specimen
3. Record solicited symptoms, AEs, and concomitant medication use
4. Inquire about MA-LRIs, SAEs, and SNMCs

Unscheduled Illness Visit 29 days or later after a dose of study vaccine

Visit PRN

1. Perform targeted physical exam
2. Collect nasal wash specimen if illness visit is associated with MA-LRI, respiratory SAE, or any related SAE (assessed as possibly, probably, or definitely related to study vaccine)
3. Inquire about MA-LRIs, SAEs, and SNMCs

3.5 Subject Evaluation Methods

3.5.1 Safety Evaluations

Each subject's legal representative will be given a thermometer and daily assessment worksheets with instructions. Beginning on the evening of each study vaccine dosing and for the following 28 days (0-28 days post each dose), the subject's legal representative will record daily temperatures (axillary route preferred), the occurrence of solicited symptoms, other AEs, and concomitant medication use. The temperature should be taken at approximately the same time each day. If more than one temperature is taken on the same day using the same route, the highest

value should be recorded. Study staff will collect and review study worksheet information and resolve any discrepancies at study site visits.

AEs that are ongoing at 28 days after any dose will be followed until resolution or stabilization by weekly telephone contacts for up to 6 additional weeks and, if needed, by monthly contacts through 365 days post randomization.

The study staff will contact the subject's legal representative by telephone on a monthly basis after the subject has completed the final scheduled clinic visits to assess the occurrence of SAEs, MA-LRIs and SNMCs. A final telephone contact is scheduled for 365 days after randomization. The subject's legal representative will be instructed to notify study personnel at any time during the study if the subject is hospitalized or has any unusual, alarming, or unexpected events.

Solicited symptoms, AEs, MA-LRIs, SAEs, and SNMCs and concomitant medication use will be documented in the subject's record and entered into the CRFs.

3.5.2 Viral Shedding Evaluation

Scheduled Sample Collection

A nasal wash specimen will be collected on Days 7, 12, and 28 following each dose to assess vaccine virus replication and shedding in the upper respiratory tract.

Samples Collected in Association with an Illness Visit

Additional nasal wash specimens for illness cultures will be collected within 24 hours or as soon as possible after illness onset or health care provider visit. See Section 3.4 for list of symptoms/illnesses requiring an illness visit and APPENDIX C for definition of terms.

Methods for Sample Collection, Handling, and Testing

Once collected, nasal wash specimens should be stabilized in viral transport media. Specimens must be handled in accordance with the instructions in the Laboratory Manual. Nasal specimens will be shipped to a MedImmune-approved central virology laboratory within 24 hours of collection or as soon as possible thereafter. Any samples (from scheduled visits or illness visits) that are determined to be positive for RSV at the central virology laboratory will be sent to the MedImmune Clinical Testing Laboratory (CTL; Mountain View, CA) for further characterization (eg, identification as wild-type or vaccine-like virus, and, as appropriate, quantitation, genotyping, and/or phenotyping) and stored for potential future testing. Samples collected at illness visits will be tested for respiratory pathogens, including RSV, at the central virology laboratory to determine the etiology of the illness episode. In addition, samples collected at illness visits where the illness

qualifies as a MA-LRI will undergo rapid detection of vaccine virus as well as other respiratory viruses for additional safety monitoring purposes.

The protocol-specific procedures for collection and processing of all specimens are detailed in the Lab Manual. The appropriate sample requisition or control forms must accompany all nasal specimens sent to the central virology laboratory and MedImmune CTL for analysis.

3.5.3 Immune Response Evaluations

Two blood samples will be drawn from each subject for immunogenicity evaluation. All subjects will have blood draws for immunogenicity evaluation performed on Day 0 (prior to administration of Dose 1) and 28 days after Dose 3 (or final dose if subject prematurely terminates study dosing). Serum samples will be stored at -20°C and sent to MedImmune CTL (or a MedImmune-approved central laboratory) for immunogenicity evaluation and potential future testing.

Subjects whose legal representatives have provided written informed consent for additional sampling may have 3 additional blood draws performed for immunogenicity evaluations. Written informed consent will be obtained from the legal representative of each of these subjects prior to study entry. For subjects scheduled to have the additional blood draws performed, samples will be obtained 7 and 28 days after Dose 1 and 28 days after Dose 2.

Immune response will be evaluated by measuring functional serum antibody responses to RSV (microneutralization assay). In addition, because the immune response generated by live attenuated vaccines is poorly understood, a number of exploratory immunology assays may be conducted to guide further vaccine development. These assays may be performed according to the presence of appropriate samples and appropriate assays.

- Specific IgG and IgA antibody to RSV by investigational ELISA
- Mucosal immune responses using IgG and IgA ELISA; specific IgG and IgA will be standardized to total IgG or IgA
- Mucosal cytokine profile
- Exploratory transcription profiling (eg, mRNA microarray analysis, a tool for studying the function of genes by revealing their pattern of expression or regulation; it is not genetic testing) may be used to characterize the nature of the immune response

3.6 Completion of Study and Loss to Follow-up

Subjects will be considered to have completed the study if they were followed up through the final scheduled study contact at 365 days post randomization. It should be specified in the CRF

whether or not the subject completed the study follow-up procedures through 28 days post each dose and through the final scheduled study contact.

Subjects will be considered lost-to-follow-up only if no contact has been established by the time the study is completed such that there is insufficient information to determine the subject's status at 365 days post randomization. Investigators should document attempts to re-establish contact with missing subjects throughout the study period. If contact with a missing subject is re-established, follow-up should resume according to the protocol.

4 SAFETY ASSESSMENT

4.1 Adverse Events

4.1.1 Definition of Adverse Events

As defined by the ICH Guideline for Good Clinical Practice (CPMP/ICH/135/95), an Adverse Event (AE) is:

> Any untoward medical occurrence in a participant or clinical investigations subject administered a pharmaceutical product and which does not necessarily have to have a causal relationship with this treatment. An adverse event (AE) can therefore be any unfavorable and unintended sign (including an abnormal laboratory finding), symptom, or disease temporally associated with the use of a medicinal product, whether or not considered related to the medicinal product.

Any adverse change from the study subject's baseline condition that occurs following the first administration of study product is an adverse event. This includes the occurrence of a new adverse event or the worsening of a baseline condition, whether or not considered related to the study product. Adverse events include but are not limited to adverse changes in the general condition of the study subject; signs and symptoms noted by the study subject or his/her care giver; concomitant disease with onset or increased severity after the start of study product administration and clinically meaningful adverse changes in laboratory safety parameters occurring after the start of study product administration. Day-to-day fluctuations in pre-existing conditions that represent a clinically significant change in the subject's status should be reported as adverse events.

4.1.2 Definition of Solicited Symptoms

Solicited symptoms are predefined symptoms or events to be specifically inquired about and assessed daily during the 28-day period after vaccine administration. Solicited symptoms will be

recorded in the CRF any time they are observed by the subject's legal representative or study staff, even in the presence of an underlying diagnosis and even if the symptom was present prior to dosing.

The solicited symptoms for this study include:

- Fever ≥100.4°F (≥38.0 °C) regardless of route (axillary route preferred for daily measurements)
- Runny/stuffy nose
- Cough
- Drowsiness
- Loss of appetite/decreased urine output
- Irritability/Fussiness
- Oropharyngeal irritation (laryngitis)
- Epistaxis

4.1.3 Definition of Medically Attended Lower Respiratory Illness

A medically attended lower respiratory illness (MA-LRI) is defined as a health care provider confirmed diagnosis of any one or more of the following events (see APPENDIX C for a full definition of terms):

- Wheezing
- Pneumonia
- Croup (laryngotracheobronchitis)
- Rhonchi (not cleared with cough or suctioning)
- Rales (not cleared with cough or suctioning)
- Bronchitis
- Bronchiolitis
- Apnea

4.1.4 Definition of Significant New Medical Conditions

A significant new medical condition (SNMC) is a newly diagnosed medical condition that is of a chronic, ongoing nature and is assessed by the investigator as medically significant. Examples of SNMCs include but are not limited to diabetes, asthma, autoimmune disease (eg, lupus, rheumatoid arthritis), and neurological disease (eg, epilepsy, autism). Events that would not be

considered SNMCs are mild eczema, diagnosis of a congenital anomaly present at study entry, or acute illness (eg, otitis media, bronchitis).

4.1.5 Study Reporting Period for Solicited symptoms, AEs, MA-LRIs, and SNMCs

The reporting period for solicited symptoms and unsolicited AEs is the period immediately following each study vaccine administration through 28 days after each dose. AEs must be followed until resolution or stabilization, even if this extends beyond the 28-day post-dosing reporting period (for the purpose of obtaining a stop date for the event).

The reporting period for SNMCs is the period immediately following initial study vaccine administration through 365 days post randomization.

The reporting period for MA-LRIs is the period immediately following initial study vaccine administration through 365 days post randomization. An MA-LRI is also an **immediately reportable event (**see Section 4.5).

4.1.6 Study Reporting Period for Enhanced Disease Surveillance

Although there is no evidence that vaccination with any live attenuated RSV candidates predisposes subjects to increased severity of disease with subsequent RSV infections, the goal of this surveillance is to evaluate the link between any severe morbidity/hospitalization and RSV infections. To monitor for possible enhanced RSV disease, laboratory confirmation of the presence or absence of RSV in respiratory secretions will be collected for all MA-LRIs. All MA-LRIs will be assessed for severity according to APPENDIX B. The overall grading of a MA-LRI should be the highest degree of severity assigned among all of the components of the respiratory event detailed in APPENDIX B. Nasal samples will be collected by the investigator within 24 hours (or as soon as possible) after onset of the event and will be analyzed at a central laboratory for respiratory virus identification. The reporting period for enhanced disease surveillance is the period immediately following study vaccine administration through 365 days post randomization. In addition, information regarding oxygenation status, supplemental oxygen requirements and specific drug treatment will be collected, as well as any other concurrent respiratory illness present at the time of the diagnosis of the event.

In the unlikely event that a statistically significant difference between vaccine group and placebo group in the rate of RSV-related hospitalization or death at the end of the study follow-up period (365 days after randomization in a cohort) is observed, then the protocol and informed consent form will be amended to continue to follow enrolled subjects through an additional RSV season. For the purpose of surveillance, the following definition for RSV enhanced disease will be used: a clinically significant increase in rates of RSV-related hospitalization or death in vaccinees

compared with placebo subjects. To qualify as an RSV-related hospitalization or death, either wild-type RSV must have been identified in a clinical specimen OR a valid negative result for RSV testing was not available from a clinical specimen obtained during the window from 2 days prior to onset of symptoms to 5 days after onset of symptoms (ie, in the absence of a confirmed negative culture, events are analyzed as if associated with shedding of wild-type RSV).

4.1.7 Recording of Solicited symptoms, AEs, MA-LRIs and SNMCs

AEs, MA-LRIs and SNMCs will be reported on the CRF using a recognized medical term or diagnosis that accurately reflects the event. Solicited symptoms will be reported on the CRF using the pre-defined terms in this protocol and will be assessed by severity. AEs and SAEs will be assessed by the investigator for severity, relationship to study vaccine, possible etiologies, and whether the event meets criteria as a Grade 3/4 AE, SAE, or a MA-LRI and therefore requires immediate notification of the sponsor (Sections, 4.2.3, 4.2.4 and 4.2.5). SNMCs will be assessed for relationship to study vaccine. See Sections 4.3 and 4.4 regarding guidelines for assessment of severity and relationship, respectively, Section 4.2.1 for the definition of SAEs, and Section 4.1.3 for the definition of MA-LRIs. If an AE has not resolved at the end of the study reporting period it will be documented as ongoing on the corresponding CRF. If an AE or solicited symptom evolves into a condition that becomes "serious" it will be reported on the Serious Adverse Event Report Form. Information recorded on the Adverse Event Case Report Form must be substantiated in the source documents.

4.2 Serious Adverse Events

4.2.1 Definition of Serious Adverse Events

A Serious Adverse Event (SAE) is any adverse event that results in any of the following outcomes:

- Death
- Life-threatening*
- Inpatient hospitalization or prolongation of existing hospitalization‡
- Persistent or significant disability or incapacity†
- Congenital anomaly/birth defect (in the offspring of a subject)
- An important medical event that may not result in death, threaten life or require hospitalization may be considered a serious adverse event when, **based upon appropriate medical judgment**, it may jeopardize the subject and may require medical or surgical intervention to prevent one of the outcomes listed above. Examples of such medical events include allergic bronchospasm requiring intensive treatment in an emergency room

or at home, blood dyscrasias or convulsions that do not result in inpatient hospitalization, or the development of drug dependency or drug abuse.

*Life threatening: An adverse event is life threatening if the subject was at risk of death at the time of the event; it does not refer to an event which hypothetically might have caused death if it were more severe.

‡Hospitalization: An inpatient hospitalization will be defined as an admission (or emergency room visit) for a period greater than 24 hours. Hospitalization for either elective surgery related to a pre-existing condition which did not increase in severity or frequency following initiation of the study or for routine clinical procedures¶ (including hospitalization for "social" reasons) that are not the result of an adverse event need not be considered as adverse events and are therefore not serious adverse events.

¶Routine Clinical Procedure: A procedure which may take place during the study period and should not interfere with the study product administration or any of the ongoing protocol specific procedures. If anything untoward is reported during an elective procedure, that occurrence must be reported as an adverse event or serious adverse event, according to the usual criteria.

†Disabling/incapacitating: An adverse event is incapacitating or disabling if the event results in a substantial disruption of the subject's ability to carry out normal life functions. This definition is not intended to include experiences of relatively minor medical significance such as headache, nausea, vomiting, diarrhea, influenza, and accidental trauma (eg, sprained ankle).

4.2.2 Study Reporting Period for Serious Adverse Events

The reporting period for serious adverse events is the period immediately following the first administration of study vaccine through 365 days post randomization. Serious adverse events must be followed until resolution by the investigator, even if this extends beyond the study reporting period. Resolution of a serious adverse event is defined as the return to baseline status or stabilization of the condition with the expectation that it will remain chronic.

At any time after completion of the study, if an investigator becomes aware of a serious adverse event that is suspected by the investigator to be related to study product, the event should be reported to MedImmune Product Safety.

4.2.3 Notification of Sponsor of Serious Adverse Events

Within 24 hours of identifying a serious adverse event, regardless of the presumed relationship to the study product, the investigator must complete the SERIOUS ADVERSE EVENT (SAE) report form and fax it to MedImmune Product Safety (or designee).

Note: Provide all available SAE information at the time of form completion. When additional information becomes available, the investigator will submit a follow-up SAE Report Form via fax to MedImmune Product Safety

MedImmune contact information:

Product Safety
MedImmune
[redacted]
Fax: [redacted]

MedImmune, as sponsor of the study being conducted under an Investigational New Drug Application (IND), is responsible for reporting certain serious adverse events as IND safety reports to the FDA, other applicable regulatory authorities, and participating investigators, in accordance with the U.S. Code of Federal Regulations (21 CFR 312.32 and 312.33), ICH Guidelines, and/or local regulatory requirements. MedImmune may be required to report certain serious adverse events to regulatory authorities within 7 calendar days of being notified about the event; therefore, it is important that investigators submit additional information requested by MedImmune as soon as it becomes available.

4.2.4 Notification of Institutional Review Board or Independent Ethics Committee of Serious Adverse Events

The investigator must comply with the applicable regulatory requirements related to the reporting of serious adverse events to the Institutional Review Board or Independent Ethics Committee. The Institutional Review Board or Independent Ethics Committee must be informed in a timely manner by the principal investigator of serious adverse events occurring at their site during the study. Investigators must also submit safety information provided by MedImmune to the Institutional Review Board or Independent Ethics Committee as detailed in Section 7.2.

4.2.5 Recording of Serious Adverse Events

Serious adverse events will be recorded on the Serious Adverse Event (SAE) Report Form using a recognized medical term or diagnosis that accurately reflects the event. Serious adverse events will be assessed by the investigator for severity, relationship to the study product and possible etiologies. See Sections 4.3 and 4.4 regarding guidelines for assessment of severity and relationship, respectively. For the purposes of study analysis, if the event has not resolved at the end of the study reporting period it will be documented as ongoing on the Adverse Event Case Report Form. For purposes of regulatory safety monitoring the investigator is required to follow

the event to resolution and report to the sponsor the outcome of the event using the Serious Adverse Event (SAE) Report Form.

4.3 Assessment of Severity

Assessment of severity is one of the responsibilities of the investigator in the evaluation of solicited symptoms, AEs, and SAEs. The determination of severity should be made by a health care professional who is qualified to review adverse event information, provide a medical evaluation of adverse events, and classify adverse events based upon medical judgment and the severity categories of *Grade 1* (*Mild*), *Grade 2* (*Moderate*), *Grade 3* (*Severe*), and *Grade 4* (*Potentially Life Threatening*) as provided in APPENDIX A and APPENDIX B.

4.4 Assessment of Relationship

The investigator is required to provide an assessment of relationship of solicited symptoms, AEs, MA-LRIs, SNMCs, and SAEs to the study product. A number of factors should be considered in making this assessment including: 1) the temporal relationship of the event to the administration of study product; 2) whether an alternative etiology has been identified; and 3) biological plausibility. The following guidelines should be used by investigators to assess the relationship of an adverse event to study product administration.

Relationship assessments that indicate an **“Unlikely Relationship”** to study product:

None: The event is related to an etiology other than the study product (the alternative etiology must be documented in the study subject’s medical record).

Remote: The event is unlikely to be related to the study product and likely to be related to factors other than study product.

Relationship assessments that indicate a **“Likely Relationship”** to study product:

Possible: There is an association between the event and the administration of the study product and there is a plausible mechanism for the event to be related to study product; but there may also be alternative etiology, such as characteristics of the subject’s clinical status or underlying disease.

Probable: There is an association between the event and the administration of study product, a plausible mechanism for the event to be related to the study product and the event could not be reasonably explained by known characteristics of the subject’s clinical status or an alternative etiology is not apparent.

Definite: There is an association between the event and the administration of study product, a plausible mechanism for the event to be related to the study product and causes other than the study product have been ruled out and/or the event re-appeared on re-exposure to the study product.

4.5 Other Events Requiring Immediate Reporting

In addition to SAEs, the following immediately reportable events (IREs) must be entered into the appropriate CRF page by the investigator ***within 24 hours***:

1. Any withdrawal of consent during the study after the subject is randomized
2. Any Grade 3 or Grade 4 AE
3. Any MA-LRI (see Section 4.1.3 and APPENDIX C for definition)
4. Permanent discontinuation of study vaccine administration

Note: The investigator is responsible for completing the Safety Fax Notification/IRE Report Form and faxing it to MedImmune Product Safety within 24 hours.

4.6 Safety Management During the Study

The MedImmune Medical Monitor has primary responsibility for the ongoing medical review of safety data throughout the study. This includes immediate review of SAEs, MA-LRIs, and Grade 3/Grade 4 AEs, which are all immediately reportable events, and timely review of other AEs reported during the study. Monthly data listings of preliminary safety data will be provided for review of events occurring within 28 days of any study dose. The Medical Monitor will determine if the threshold has been met for immediate review of Grade 3/Grade 4 AEs (see Section 4.8), SAEs, and MA-LRIs by the Unblinded Safety Physician and Safety Biostatistician (a clinical trial specialist and statistician not directly associated with the conduct of the study). Once the threshold has been met for review of an event(s), the Unblinded Safety Physician and Safety Biostatistician have primary responsibility for assessment of Grade 3/Grade 4 AEs, MA-LRIs, and SAEs to determine whether stopping rules have been met such that events must be reviewed by the SMC (see below). At their discretion, they may review blinded or unblinded data in listings or tables, including virology data.

Consistent with protocol Section 4.1.6 (Study Reporting Period for Enhanced Disease Surveillance), the Medical Monitor has responsibility for ongoing review of MA-LRI events through 365 days post dosing to assess the need for review by the Unblinded Physician and Safety Biostatistician. For this purpose, all MA-LRI hospitalizations or deaths (whether occurring within or later than 28 days post dosing) will be attributed, hypothetically, to the vaccine arm, and if this would result in a statistical imbalance in these events, the Unblinded Physician and Safety

Biostatistician will review events in blinded or unblinded fashion in conjunction with RSV testing and event severity data to determine if imbalance has occurred. The Medical Monitor can also request review by the Unblinded Physician and Safety Biostatistician if concerns arise regarding potential imbalance in rates of MA-LRI events or severity of MA-LRI events.

If statistical imbalance in all MA-LRI hospitalizations or deaths through 365 days after randomization has occurred or if otherwise requested by the Unblinded Safety Physician and Safety Statistician, the SMC will review all MA-LRI events for a decision regarding stopping enrollment temporarily to gather additional information, stopping enrollment permanently, or amending protocol and informed consent to follow subjects through an additional RSV season.

The Medical Monitor also has the responsibility of reviewing safety data prior to initiation of Cohort 2 dosing and after enrollment of the first 40 subjects in Cohort 2 (see Section 4.9).

The MedImmune Product Safety Specialist has responsibility for the receipt, review, investigation, and follow-up of SAEs reported by the clinical study sites.

The SMC is composed of at least two MedImmune physicians who are not directly involved in the day-to-day operations of the study, and at least two physicians who are not employees of MedImmune. The SMC will independently review cumulative safety surveillance data prior to initiation of Cohort 2 dosing and after enrollment of the first 40 subjects in Cohort 2 (see Section 4.9). The SMC will also review safety data at other times in response to SAEs, MA-LRIs, Grade 3/Grade 4 AEs, or other AEs considered medically significant by the Medical Monitor or the Unblinded Safety Physician and Safety Biostatistician (see Section 4.8). Additionally, the SMC reviews immediately reportable events monthly. The SMC will review the data in a treatment blinded or unblinded manner and determine whether randomization and/or dosing should be discontinued. SMC review is required for resumption of the study in the event that study randomization and/or dosing are stopped according to stopping rules in Section 4.8. Additionally, the SMC will review decisions of the Medical Monitor and the Unblinded Safety Physician and Safety Biostatistician regarding study vaccination on a regular basis throughout the study. When SMC decisions regarding discontinuation or resumption of dosing and/or randomization are made, these decisions will be communicated promptly to the FDA and to other regulatory authorities as applicable.

The SMC conducts all safety assessment independently of the Medical Monitor. Closed sessions are held as necessary to review blinded data and to reach consensus without the presence of the Medical Monitor. The SMC decision is the final decision and supersedes the assessment of the Medical Monitor or Unblinded Safety Physician and Biostatistician.

4.7 Interruption or Discontinuation of Study Dosing in Individual Subjects

An individual subject will not receive a scheduled study vaccine dose if any of the following occur in the subject in question. The subject may be vaccinated at a later date *upon resolution* of the event provided that vaccination occurs within the protocol-specified vaccination window.

1. Any fever (≥100.4°F [≥38.0°C]), regardless of route, at the time of study vaccine administration
2. Acute illness at time of study vaccine administration (acute illness is defined as the presence of moderate or severe signs and symptoms)
3. Moderate or severe nasal congestion that in the investigator’s opinion could prevent intranasal delivery of the study vaccine

An individual subject will not receive a scheduled study vaccine dose if any of the following occur in the subject in question. The subject may be vaccinated at a later date provided that vaccination occurs within the protocol-specified vaccination window.

1. Receipt of any live virus vaccine (excluding rotavirus vaccine) within 28 days prior to vaccination or expected receipt within a 28 day window around any dose
2. Receipt of any inactivated (eg, non-live) vaccine or rotavirus vaccine within 14 days prior to vaccination or expected receipt within a 14 day window around any dose
3. Recent use or expected receipt of immunosuppressive agents including steroids (≥2 mg/kg per day of prednisone or its equivalent, or ≥20 mg/day if the subject weighs >10 kg, given daily or on alternate days for ≥14 days); children in this category should not receive study vaccine until immunosuppressive agents including corticosteroid therapy have been discontinued for ≥30 days
4. Living in the same home or enrolled in the same classroom at full-time day care with infants <6 months of age within 28 days after each dose
5. Contact with pregnant caregiver within 28 days after each dose
6. Living in a household with someone who is immunocompromised within 28 days after each dose; the subject should also avoid close contact with immunocompromised individuals for 28 days after each dose
7. Living in a household with someone who works in the healthcare field and who has direct patient care responsibilities within 28 days after each dose
8. Living in a household with someone who is a day care provider or preschool teacher for children <6 months of age within 28 days after each dose
9. Any condition that, in the opinion of the investigator, might interfere with study vaccine evaluation

If a subject does not receive study vaccine during the Dose 2 vaccination window, the subject may receive study vaccine in the Dose 3 vaccination window, if eligible. If Dose 2 is missed, the Dose 3 vaccination window will be 96 to 120 days post Dose 1. Unless consent is withdrawn, subjects who receive 1, 2, or 3 doses of study vaccine will be followed for the full study period through 365 days post randomization, including the collection of any protocol-specified samples (final blood sample and nasal wash samples in accordance with APPENDIX C). Subjects who miss a dose of vaccine will not have the post-dose study visits and nasal washes performed. Subjects who have dosing interrupted in accordance with Section 4.7 (Interruption or Discontinuation of Study Dosing in Individual Subjects) and who are expected to receive a subsequent dose of vaccine will not have blood obtained after dosing. Subjects who have study vaccine permanently discontinued (see Subsection, Permanent Discontinuation of Study Vaccine) should have a study visit to obtain a final post-dosing blood sample 28 to 34 days post last dose or within 4 weeks of the decision to discontinue all dosing. For all subjects, unscheduled visits to obtain illness nasal washes must be held in accordance with APPENDIX C.

Permanent Discontinuation of Study Vaccine

An individual subject will not receive any further study vaccine if any of the following occur in the subject in question:

1. Withdrawal of consent
2. Acute allergic reaction (consistent with a significant IgE mediated event) or anaphylaxis following the administration of a previous dose
3. Any respiratory SAE
4. Any SAE assessed as related (possibly, probably, or definitely) to study vaccine
5. Use of any investigational or non-registered drug or vaccine product since the previous dose administration
6. Intercurrent history of receipt of blood transfusion or expected receipt through 28 days following final study vaccine dosing
7. Intercurrent history of receipt of immunoglobulin products or expected receipt through 28 days after final study vaccine dosing
8. Event which in the opinion of the investigator or Medical Monitor contraindicates further dosing such as illnesses or complications
9. Noncompliance with protocol that, in the opinion of the investigator or sponsor, limits the ability to follow the subject for safety or to obtain trial data
10. Termination of research by sponsor or investigator
11. Known or suspected immunodeficiency including HIV

Unless consent is withdrawn, subjects who are permanently discontinued from study vaccine will be followed (including the collection of all protocol-specified samples) for the full study period (through 365 days after randomization). Subjects who are permanently discontinued from study vaccine should have a blood sample obtained 28 to 34 days post last dose or as soon as possible once the decision is made to discontinue all dosing, and visits to obtain illness nasal washes must be held in accordance with APPENDIX C.

4.8 Interruption or Discontinuation of Overall Study Dosing and Randomization

Interruption or Discontinuation of Overall Study Dosing and Randomization

If any of the following occur, no further administration of study vaccine will take place, and no further subjects will be randomized into the study until review of the event in question by the Medical Monitor and the SMC:

1. Any SAE assessed as related (possibly, probably or definitely, per definitions in Section 4.4) to study vaccine
2. Any event that, in the opinion of the investigator, Medical Monitor, or Unblinded Safety Physician, contraindicates dosing of additional subjects

Interruption or Discontinuation of Study Randomization

If any of the following occur, the IVRS system will be locked and no additional subjects will be randomized into the study until a review of the event in question is performed by the Medical Monitor and the SMC:

1. Any MA-LRI associated with shedding of vaccine virus whether or not another respiratory pathogen has been detected

Note: If viral culture results (or, in the event that culture results are not available for technical reasons, PCR results) are not available for a nasal wash specimen collected between 2 days prior to onset through 5 days after onset, an MA-LRI event will be assessed as if associated with shedding of vaccine virus, if the MA-LRI occurs within 28 days post dose

2. The occurrence of a statistically significant imbalance (analyzed by subject) either cumulatively (ie, among all cohorts) or by cohort in Grade 3 and 4 adverse events (includes MA-LRI events) occurring within 28 days after dosing with more events in vaccine arm compared to placebo arm

3. Any event, which in the opinion of the investigator, Medical Monitor, or Unblinded Safety Physician contraindicates enrollment of additional subjects

If study randomization has been interrupted pending SMC review of event(s) while other study dosing continues, and if the SMC has found that randomization should not be re-instituted, all

study dosing also must be discontinued, unless the SMC has specifically determined that dosing may continue.

4.9 Cohort Progression

The target sample size for Cohort 1 is 160 children, 5 to <24 months of age, randomized 1:1 to receive MEDI-559 or placebo on a 0, 2, and 4 month schedule. All subjects will be pre-screened and only subjects who are seronegative for RSV will be enrolled. Cohort 1 will initiate dosing at 10^5 FFU MEDI-559. The Medical Monitor and SMC will review blinded safety data from the first 40 subjects enrolled in Cohort 1 for the 28 days following administration of the first dose of vaccine. Safety data for review includes solicited symptoms, AEs, SAEs, and MA-LRIs. The SMC may also choose to review data by A/B grouping (treatment A vs. treatment B) or by unblinded grouping (MEDI-559 vs. placebo). If no safety concerns are noted, Cohort 2 will initiate dosing at 10^5 FFU MEDI-559.

The target sample size for Cohort 2 is 160 infants, 1 to <3 months of age, randomized 1:1 to receive either MEDI-559 or placebo on a 0, 2, and 4 month schedule. Subjects in Cohort 2 will not be screened for RSV serostatus prior to enrollment, because it is expected that these infants will have passively acquired maternal antibody.

Enrollment into Cohort 2 will be halted after the enrollment of approximately 40 subjects. The Medical Monitor and SMC will review available blinded safety data through 28 days post Dose 1 for these subjects. Safety data for review includes solicited symptoms, AEs, SAEs, and MA-LRIs. The SMC may choose to review data by A/B grouping (treatment A vs. treatment B) or by unblinded grouping (MEDI-559 vs. placebo). If no safety concerns are noted, the remainder of Cohort 2 will be enrolled. During the time that enrollment into Cohort 2 is halted for safety assessment, previously enrolled subjects should receive Dose 2 or Dose 3 within the appropriate protocol-specified dosing window.

4.10 Monitoring of Dose Administration

As with any vaccine, allergic reactions to dose administration are possible. Therefore, appropriate drugs and medical equipment to treat acute anaphylactic reactions must be immediately available, and study personnel must be trained to recognize and treat anaphylaxis. All subjects will be observed at the study site for a minimum of 30 minutes following dosing. Emergency management supplies (eg, AMBU bag, adrenaline [epinephrine], antihistamines) must remain available for initial treatment of an allergic reaction if needed.

5 STATISTICAL CONSIDERATIONS

5.1 General Considerations

Data will be provided in listings sorted by cohort, treatment group, and PID. Tabular summaries will generally be presented by cohort and treatment group. Categorical data will be summarized by the number and percentage of subjects in each category. Continuous variables will be summarized by descriptive statistics including mean, standard deviation, median, minimum, and maximum. No formal statistical tests are planned.

Missing data will be treated as missing. No data will be imputed.

5.2 Sample Size

Sample size was based on clinical rather than statistical considerations.

In each cohort, 80 subjects are planned to receive MEDI-559, which should be a sufficient number to understand how the vaccine behaves in the corresponding age group. This number of subjects should also be sufficient to support the future proof-of-concept study.

Table 2 provides 95% confidence intervals (CIs) for a range of potential differences in observed event rates for each cohort. Table 3 provides the probability of observing at least one event with 80 MEDI-559 recipients, assuming various underlying event rates. The probability of observing at least one event with 80 subjects will be 55%, 98%, or >99% when the true rate is 1%, 5%, or 10%, respectively.

Table 2 Differences in Observed Event Rate and Corresponding 95% CIs for each Cohort

MEDI-559 (N=80)	Placebo (N=80)					
	0% (n=0)	5% (n=4)	10% (n=8)	20% (n=16)	30% (n=24)	40% (n=32)
0% (n=0)	0% (-4.5%, 4.6%)	-5% (-12.3%, -0.2%)	-10% (-18.8%, -4.0%)	-20% (-30.4%, -11.9%)	-30% (-41.3%, -20.3%)	-40% (-51.6%, -29.2%)
5% (n=4)	5% (0.2%, 12.3%)	0% (-7.9%, 7.9%)	-5% (-14.3%, 3.6%)	-15% (-25.9%, -4.0%)	-25% (-36.8%, -12.9%)	-35% (-47.0%, -22.5%)
10% (n=8)	10% (4.0%, 18.8%)	5% (-3.6%, 14.3%)	0% (-10.0%, 10.0%)	-10% (-21.7%, 1.2%)	-20% (-32.5%, -6.0%)	-30% (-42.7%, -16.0%)
20% (n=16)	20% (11.9%, 30.4%)	15% (4.0%, 25.9%)	10% (-1.2%, 21.7%)	0% (-12.8%, 12.8%)	-10% (-23.5%, 3.6%)	-20% (-34.1%, -5.3%)
30% (n=24)	30% (20.3%, 41.3%)	25% (12.9%, 36.8%)	20% (6.0%, 32.5%)	10% (-3.6%, 23.5%)	0% (-14.8%, 14.8%)	-10% (-24.7%, 4.9%)
40% (n=32)	40% (29.2%, 51.6%)	35% (22.5%, 47.0%)	30% (16.0%, 42.7%)	20% (5.3%, 34.1%)	10% (-4.9%, 24.7%)	0% (-15.3%, 15.3%)
50% (n=40)	50% (38.6%, 61.4%)	45% (32.5%, 57.0%)	40% (25.9%, 52.8%)	30% (15.1%, 43.7%)	20% (4.7%, 34.7%)	10% (-5.7%, 25.4%)

Produced in StatXact per exact method of Chan and Zhang (Chan, 1999).

Table 3 Probability of Observing ≥1 Event for N=80 MEDI-559 Subjects

True Event Rate (N=80)	Probability of Observing at Least One SAE
1%	55%
2%	80%
3%	91%
4%	96%
5%	98%
10%	>99%

5.3 Subject Populations

All randomized subjects who receive any study vaccine and have safety data available will be included in safety summaries.

Vaccine virus shedding summaries will include all randomized subjects who receive study vaccine and do not have a major protocol violation that would affect interpretation of the shedding results and for whom at least one shedding sample is available.

Immune response summaries will include all randomized subjects who receive study vaccine and do not have a major protocol violation that would affect interpretation of immune response assay results and for whom at least one assay result is available.

5.4 Primary Endpoints

The primary endpoints of safety and tolerability of MEDI-559 will be measured by:

- Incidence of solicited symptoms from administration of study vaccine through 28 days following each dose
- Incidence of adverse events from administration of study vaccine through 28 days following each dose
- Incidence of MA-LRIs from administration of study vaccine through 28 days following each dose
- Incidence of SAEs from administration of study vaccine through 28 days post final dose

5.5 Secondary Endpoints

The secondary endpoints include the following:

- Incidence and magnitude of MEDI-559 shedding at Days 7, 12, and 28 after each dose
- Percentage of subjects with a seroresponse to RSV after the final dose (defined as a ≥4-fold rise from baseline in neutralizing antibody titer, regardless of baseline serostatus)
- Description of the genotypic and phenotypic stability of recovered vaccine virus
- Description of the potential for immunologic enhancement of RSV disease by evaluation of MA-LRIs associated with RSV infection through 365 days after randomization
- Incidence of SAEs from administration of study vaccine through 365 days after randomization
- Description of SNMCs from administration of study vaccine through 365 days after randomization

5.6 Other Analyses

Data on rotavirus vaccine administration will be prospectively collected and the effects of rotavirus vaccine administration, relative to MEDI-559 administration, AEs, shedding, and immunogenicity will be explored. Data will also be summarized for any subjects who do not receive one or more of their scheduled rotavirus vaccinations.

This study will be of sufficient size to explore the immune response generated by MEDI-559. A number of experimental immunological read-outs will be evaluated to help guide future product development (see Section 3.5.2)

Other exploratory assays may be performed according to the presence of appropriate samples and appropriate assays.

6 DATA HANDLING AND MONITORING

The primary source document for this study will be the subject's medical record. If separate research records are maintained by the investigator(s), both the medical record and the research records will be considered the source documents for the purposes of auditing the study.

Data recorded on source documents will be captured on case report forms and will be reviewed by the sponsor or designee. A copy of each completed case report form will be retained by the investigator.

The study will be monitored by MedImmune or its designee on a regular basis throughout the study period. All study documents (subject files, signed informed consent forms, copies of case report forms, Study File Notebook, etc.) must be kept secured for a period of 2 years following marketing of MEDI-559 or for 2 years after centers have been notified that the IND has been discontinued. There may be other circumstances for which MedImmune is required to maintain study records and, therefore, MedImmune should be contacted prior to removing study records for any reason.

7 HUMAN SUBJECTS

7.1 Ethics and Regulatory Considerations

The study will be conducted according to the Declaration of Helsinki, Protection of Human Volunteers (21 CFR 50), Institutional Review Boards (21 CFR 56), and Obligations of Clinical Investigators (21 CFR 312).

The protocol will be reviewed and approved by the Institutional Review Board (IRB) or Independent Ethics Committee (IEC) of each participating center prior to study initiation. Serious adverse events regardless of causality will be reported to the sponsor and to the IRB/IEC, and the investigator will keep the IRB/IEC informed as to the progress of the study.

The investigator will explain the nature of the study and will inform the subject's legal representative that participation is voluntary and that they can withdraw their child at any time. Written informed consent will be obtained from each subject's legal representative prior to entry into the study. A copy of the signed consent form will be given to every subject's legal representative and the original will be maintained with the subject's records.

To maintain confidentiality, all laboratory specimens, evaluation forms, reports and other records will be identified by a coded number and initials only (or in accordance with local confidentiality rules). All study records will be kept in a locked file cabinet and code sheets linking a subject's name to a participant's identification number will be stored separately in another locked file cabinet. Clinical information will not be released without written permission of the subject's legal representative, except as necessary for monitoring by the FDA or other regulatory authorities or by the sponsor of the clinical trial. The principal investigator must also comply with all applicable privacy regulations (e.g. Health Insurance Portability and Accountability Act of 1996, European Union Data Protection Directive 95/46/EC).

7.2 Institutional Review Board (IRB) or Independent Ethics Committee (IEC)

A list of IRB/IEC members or an IRB assurance number should be obtained by the investigator and provided to the sponsor.

Any documents that the IRB/IEC may need to fulfill its responsibilities, such as protocol amendments, and information concerning subject recruitment, payment or compensation procedures, or information from the sponsor will be submitted to the IRB/IEC. The IRB/IEC's written unconditional approval of the study protocol and the informed consent form will be in the possession of the investigator and the sponsor before the study is initiated. The IRB/IEC's unconditional approval statement will be transmitted by the investigator to the sponsor prior to shipment of study drug supplies to the site. This approval must refer to the study by exact protocol title and number, and should identify the documents reviewed and the date of review.

Protocol modifications or changes may not be initiated without prior written IRB/IEC approval except when necessary to eliminate immediate hazards to the subjects or when the change(s) involves only logistical or administrative aspects of the study. Such modifications will be submitted to the IRB/IEC and written verification that the modification was submitted should be obtained.

The IRB/IEC must be informed by the principal investigator of informed consent changes or revisions of other documents originally submitted for review; serious and/or unexpected adverse experiences occurring during the study; new information that may affect adversely the safety of the subjects or the conduct of the study; an annual update and/or request for re-approval; and when the study has been completed.

7.3 Informed Consent

The principles of informed consent in the current edition of the Declaration of Helsinki should be implemented before any protocol-specified procedures or interventions are carried out. Informed consent will be obtained in accordance with 21 CFR 50.25.

Information should be given in both oral and written form, and subject's legal representatives must be given ample opportunity to inquire about details of the study. The written consent document will embody the elements of informed consent as described in the Declaration of Helsinki and will also comply with local regulations.

The subject's legal representatives must be informed about the aims, expected benefits, and possible risks that are currently unforeseeable. They must also be informed of alternative procedures. Subject's legal representatives must receive an explanation as to whether any compensation and any medical treatments are available if injury occurs and, if so, what they consist of, or where further information may be obtained. They must be informed whom to contact for answers to any questions relating to the research project. The subject's legal representatives must be informed that their child's participation is voluntary and that they are free to withdraw their child from the study for any reason at any time, without penalty or loss of benefits to which their child is otherwise entitled. The extent of the confidentiality of subject's records must be defined, and subject's legal representatives must be informed that applicable data protection legislation will be complied with. Subject's legal representatives must be informed that the monitor(s), auditor(s), IRB/IEC members, and the regulatory authorities will be granted direct access to the subject's original medical records for verification of clinical trial procedures and/or data, without violating the confidentiality of the subject, to the extent permitted by the applicable laws and regulations and that, by signing a written informed consent form, the subject's legal representative is authorizing such access.

The consent form generated by the investigator must be approved by the IRB/IEC and be acceptable to MedImmune. Consent forms must be written so as to be understood by the prospective subject or their legal representative. Informed consent will be documented by the use of a written consent form approved by the IRB/IEC and signed and dated by the subject's legal representative, and by the person who conducted the informed consent discussion. The signature confirms the consent is based on information that has been understood. Each subject's signed informed consent form must be kept on file by the investigator for possible inspection by Regulatory Authorities and/or MedImmune professional and Regulatory Compliance persons. The subject's legal representative should receive a copy of the signed and dated written informed consent form and any other written information provided to the subjects, and should receive

copies of any signed and dated consent form updates and any amendments to the written information provided to subjects.

8 STUDY COMPLETION

All materials or supplies provided by the sponsor will be returned to the sponsor upon study completion. The investigator will notify the IRB/IEC when the study has been completed.

9 PUBLICATIONS

Publication by the site of any data from this study must be carried out in accordance with the clinical study agreement.

10 CHANGES IN THE PROTOCOL

The protocol may not be modified without written approval of the sponsor. All changes to the protocol must be submitted to the FDA and IRB/IEC, and must be approved by the IRB/IEC prior to their implementation. Documentation of IRB/IEC approval must be sent to the sponsor immediately upon receipt.

Protocol Version 1.1 ([redacted]) was revised to Version 2.0 on ([redacted]). Changes to the protocol are described in APPENDIX E and are incorporated in the body of Protocol Version 2. No subjects were entered into the study under Version 1.0.

11 REFERENCES

Chan IS, Zhang Z. Test-based exact confidence intervals for the difference of two binomial proportions. Biometrics 1999;55:1202-1209.

Chin J, Magoffin RL, Shearer LA, Schieble JH, Lennette EH. Field evaluation of a respiratory syncytial virus vaccine and a trivalent parainfluenza virus vaccine in a pediatric population. Am J Epidemiol 1969;89:449-463.

Durbin AP, Karron RA. Progress in the development of respiratory syncytial virus and parainfluenza virus vaccines. Clin Infect Dis 2003;37:1668-1677.

Hall CB. Respiratory syncytial virus and parainfluenza virus. N Engl J Med 2001;344:1917-1928.

Karron RA, Wright PF, Crowe JE Jr, Clements-Mann ML, Thompson J, Makhene M, et al. Evaluation of two live, cold-passaged, temperature-sensitive respiratory syncytial virus vaccines in chimpanzees and in human adults, infants, and children. J Infect Dis 1997;176:1428-1436.

Karron RA, Belshe RB, Wright PF, Thumar B, Burns B, Newman F, et al. A live human parainfluenza type 3 virus vaccine is attenuated and immunogenic in young infants. Pediatr Infect Dis J 2003;22:394-405.

Karron RA Wright PF, Belshe RB, Thumar B, Casey R, Newman F, et al. Identification of a recombinant live attenuated respiratory syncytial virus vaccine candidate that is highly attenuated in infants. J Infect Dis 2005;191:1093-1104.

Lee M-S, Walker RE, Mendelman PM. Medical burden of respiratory syncytial virus and parainfluenza virus type 3 infection among US children. Human Vaccines 2005;1:6-11.

Murphy BR, Collins PL. Live-attenuated virus vaccines for respiratory syncytial and parainfluenza viruses: applications of reverse genetics. J Clin Invest 2002;110:21-27.

Wright PF, Karron RA, Belshe RB, Shi J, Randolph V, Collins, P, et al. The absence of enhanced disease with wild type respiratory syncytial virus infection occurring after receipt of live, attenuated, respiratory syncytial virus vaccines. Vaccine 2007;25:7372-7378.

Wright PF, Karron RA, Belshe RB, Thompson J, Crowe JE Jr, Boyce TG, et al. Evaluation of a live, cold-passaged, temperature-sensitive, respiratory syncytial virus vaccine candidate in infancy. J Infect Dis 2000;182:1331-1342.

APPENDIX A Severity Grading Table for Solicited Symptoms and Adverse Events

Event	Grade 1 (Mild)	Grade 2 (Moderate)	Grade 3 (Severe)	Grade 4 (Potentially Life Threatening)
**Solicited Symptoms**				
Fever (regardless of route)	100.4 – 101.4°F (38.0 – 38.5°C)	101.5 – 103.1°F (38.6 – 39.5°C)	103.2– 104.9°F (39.6 – 40.5°C)	>104.9°F (>40.5°C)
Runny/Stuffy nose	Clear or purulent nasal discharge lasting >4 hours in any 24 hour period	Clear or purulent nasal discharge that requires frequent suctioning for oral feeding or sleeping	Nasal congestion leading to respiratory distress or dehydration	Requires immediate active medical intervention to support vital functions
Cough	Cough lasting for more than 4 hours in any 24-hour period not associated with feeding/emesis, can smile and play	Cough that prevents normal feeding or prevents sleep for more than 4 hours	Cough that requires medical intervention to allow oral feeding, prevent respiratory distress or allow any sleep over a 24 hour period	Requires immediate active medical intervention to support vital functions
Drowsiness	Sleeping more than usual but can be waken easily	Somnolence prevents playing/smiling	Sleeping more than usual and difficult to arouse and prevents oral hydration	Requires immediate active medical intervention to support vital functions
Loss of appetite/ Decreased urine output	Eating or drinking less than usual for less than 1 day	Eating or drinking less than usual which leads to decrease in urine output or number of wet diapers	Does not eat/drink at all requiring NG/OG hydration or IV fluids	Requires immediate active medical intervention to support vital functions
Irritability/Fussiness	Crying more than usual but can be consoled by hugging and rocking	Crying that is difficult to console and interferes with smiling and playing	Inconsolable crying for more than 4 hours in a 24 hour period, irritability that cannot be consoled and prevents oral hydration	Requires immediate active medical intervention to support vital functions
Oropharyngeal inflammation (laryngitis)	Change in voice or cry not interfering with eating or drinking	Total loss of voice OR refuses to take solids but takes enough liquids to maintain usual urine output	Inability to swallow solid or liquid food	Requires immediate active medical intervention to support vital functions

Event	Grade 1 (Mild)	Grade 2 (Moderate)	Grade 3 (Severe)	Grade 4 (Potentially Life Threatening)
Epistaxis	Spontaneously resolves or requires <10 minutes of direct pressure	Stops after >15 minutes of direct pressure or recurs within 24 hours	Requires nasal packing or cautery to achieve hemostasis	Requires immediate active medical intervention to support vital functions
**Grading Adverse Event other than MA-LRI**				
	Transient or mild discomfort (<24 hours); no medical intervention required; symptoms cause no/minimal interference with feeding/playing	Some interference with normal activity, moderate interference with sleeping/drinking, not requiring medical intervention	Prevents daily activity and requires medical intervention	Requires significant medical attention; requires hospitalization extreme limiting of activity, medically unstable
**For Grading MA-LRIs** see APPENDIX B				

APPENDIX B Respiratory Distress Severity Grading Table

Respiratory Component	Grade 1 (Mild)	Grade 2 (Moderate)	Grade 3 (Severe)	Grade 4 (Potentially Life threatening)
Upper respiratory tract	Barky/croupy cough but able to sleep, take fluids, and play	Inspiratory upper airway sounds<br>OR barky cough that interferes with sleep	Requires systemic steroids	Requires more than 1 racemic epinephrine<br>OR hospitalization OR requires medical observation for >6 hours
Lower respiratory tract	End-expiratory wheezing	Wheezing through expiratory phase or requiring prn administration of β_2 agonists	Wheezing requiring systemic or nebulized steroids or nebulized β_2 agonists with a frequency of up to every 4 hours	Requires nebulized B_2 agonist more frequently than every 4 hours
Work of breathing	Cough lasting for more than 4 hours in any 24 hour period not associated with feed/emesis, can smile and play	Cough that prevents normal feeding or prevents sleep for more than 4 hours	Grunting,<br>OR nasal flaring,<br>OR retractions	Respiratory fatigue<br>OR respiratory rate >55 despite treatment OR<br>tachypnea despite treatment (>60 for 1-12 month old, >55 for 12-24 month old)
Oxygen saturation	Normal room air oxygen saturation 98-100%	Abnormal room-air oxygen not requiring supplemental oxygen	Room air saturation <95%	Requires supplemental oxygen to maintain oxygen saturation >93%

prn = as the circumstances require
To be implemented for scoring the severity of MA-LRIs. The overall grading of the Upper or Lower Respiratory Tract Illness should be the highest degree of severity assigned among all the components of the respiratory event.

APPENDIX C Events for Which a Nasal Wash Specimen is Required

Event/Illness	Definition
**Within 28 days after Any Dose**	
Fever	≥100.4°F (≥38.0°C), regardless of route
Cough	≥2 consecutive days of persistent cough not associated with eating or drinking
Runny/stuffy nose	≥2 consecutive days of clear or purulent discharge from the nares not associated with crying, or eating and drinking
Breathing difficulty	Increased respiratory sounds, retractions, tachypnea, color change, grunting, nasal flaring, gasping
Acute otitis media (AOM)	Loss of tympanic membrane landmarks, accompanied by erythema and loss of mobility; may or may not be associated with fever or other respiratory symptoms; confirmed with tympanometry if possible
**At Any Time during the Study (Day 0 through 365 days after randomization)**	
Wheezing*	Sustained high pitched, musical breath sounds, during the expiratory phase, which do not clear with cough and must be confirmed by auscultation with stethoscope; must not be transmitted upper airway sounds or inspiratory in nature
Pneumonia*+	Persistent rales and crackles originating in the lower respiratory tract, usually accompanied by tachypnea, which do not clear with cough; may be confirmed by x-ray showing areas of consolidation
Croup (laryngotracheobronchitis)*+	Barking cough, hoarseness, and inspiratory stridor (high-pitched whistling sound)
Rhonchi*	Persistent coarse breath sounds from the lung or large airways that are not transmitted noises from the upper airway and do not clear with cough or suctioning
Rales*	Persistent abnormal lower respiratory tract (eg, lung) sound heard through a stethoscope that does not clear with cough or suctioning; may be sibilant (whistling), dry (crackling), or wet, depending on the amount and density of fluid refluxing back and forth in the air passages
Bronchitis*	Coarse large airway rhonchi associated with productive cough; may be accompanied by other signs of respiratory infection or may be allergic
Bronchiolitis*+	Expiratory wheezing usually associated with signs and symptoms of upper respiratory tract infection, may be confirmed by x-ray showing interstitial infiltrates and hyperinflation
Apnea*	Defined by history (observed by lay person) as reported cessation of breathing for 20 seconds or greater with accompanying cyanosis pallor or collapse; breath-holding spells are excluded

All events require that a nasal specimen be collected within 24 hr or as soon as possible after the onset of illness or visit for medically attended respiratory illness.

***Event/diagnosis is deemed an immediately reportable event.** In addition, all Grade 3/Grade 4 AEs are immediately reportable events, but a nasal wash specimen is required only if the Grade 3/4 event is one of the events listed in this table.

+Final diagnosis must be confirmed by the principal investigator or through confirmatory radiograph if the initial diagnosis was made by a health care provider outside the study site.

APPENDIX D WHO Simplified Weight-Per-Age Field Tables

Female subjects should have their age and weight checked using the table below. Female subjects should not be enrolled in the study if their weight (in kg) is less than the number listed under the 5th percentile column.

Birth to 2 years (percentiles) GIRLS:

Percentiles (weight in kg)		
**Year: Month**	**Month**	**5th**
0: 0	0	2.5
0: 1	1	3.3
0: 2	2	4.1
0: 3	3	4.7
0: 4	4	5.2
0: 5	5	5.6
0: 6	6	6.0
0: 7	7	6.3
0: 8	8	6.5
0: 9	9	6.8
0:10	10	7.0
0:11	11	7.2
1: 0	12	7.3
1: 1	13	7.5
1: 2	14	7.7
1: 3	15	7.9
1: 4	16	8.1
1: 5	17	8.2
1: 6	18	8.4
1: 7	19	8.6
1: 8	20	8.7
1: 9	21	8.9
1:10	22	9.1
1:11	23	9.2
2: 0	24	9.4

Source: World Health Organization Child Growth Standards
(http://www.who.int/childgrowth/standards/weight_for_age/en/index.html)

Male subjects should have their age and weight checked using the table below. Male subjects should not be enrolled in the study if their weight (in kg) is less than the number listed under the 5th percentile column.

Birth to 2 years (percentiles) BOYS:

Percentiles (weight in kg)		
**Year: Month**	**Month**	**5th**
0: 0	0	2.6
0: 1	1	3.6
0: 2	2	4.5
0: 3	3	5.2
0: 4	4	5.8
0: 5	5	6.2
0: 6	6	6.6
0: 7	7	6.9
0: 8	8	7.2
0: 9	9	7.4
0:10	10	7.7
0:11	11	7.9
1: 0	12	8.1
1: 1	13	8.2
1: 2	14	8.4
1: 3	15	8.6
1: 4	16	8.8
1: 5	17	8.9
1: 6	18	9.1
1: 7	19	9.3
1: 8	20	9.4
1: 9	21	9.6
1:10	22	9.8
1:11	23	9.9
2: 0	24	10.1

Source: World Health Organization Child Growth Standards
(http://www.who.int/childgrowth/standards/weight_for_age/en/index.html)

APPENDIX E Summary of Changes to the Protocol

Protocol Version 1.1, [redacted]

All text revisions resulting from this version are incorporated in the body of Protocol Version 1.1.

The following changes were made in response to comments from CBER received on [redacted]:

1) Section 3.1.2 (Exclusion Criteria): Exclusion criterion #5 was revised to specify “or weight ≤5th percentile for age on the day of randomization” to be consistent with Cohort 1.
2) Section 3.3.5 (Concomitant Medications/Immunizations): The word “Immunizations” was added to the title of this section and the importance of delivering routine immunizations, including rotavirus vaccination, in accordance with the standards of care was addressed, along with a requirement that study subjects will not forego or delay the receipt of any mandatory vaccine due to study participation. In addition, it was specified that all doses of any rotavirus vaccine received by the subject from 28 days prior to the first study dose through 28 days after the final study dose be recorded.
3) Section 4.1.6 (Study Reporting Period for Enhanced Surveillance): A statement was added that the protocol and ICF would be amended to include enhanced disease surveillance through a second RSV season, if a safety signal being consistent with RSV enhanced disease is observed during the first season of follow-up. Additionally, RSV enhanced disease and any signal that would trigger a second season of enhanced disease surveillance was defined.
4) Section 5.6 (Other Analyses): Additional information was added to clarify that data on rotavirus vaccine administration will be prospectively collected for exploring the effects of rotavirus vaccine administration, relative to MEDI-559 administration, on AEs, shedding, and immunogenicity. In addition, the following statement was added “data will also be summarized for any subjects who do not receive one or more of their scheduled rotavirus vaccinations”.
5) Appendix B (Respiratory Distress Severity Grading Table): Additional wording was added to the Grade 2 and Grade 3 columns for lower respiratory tract, to include prn administration of β_2 agonists (Grade 2 column) and to specify “nebulized” steroids and β2 agonists (Grade 3 column). In addition, the description of life threatening for Grade 4 events was modified to “potentially life threatening”, and “life threatening condition” was removed in the corresponding cells of the Grade 4 column.
6) Appendix D (WHO Simplified Weight-Per-Age Field Tables): Appendix D was added to create a reference for determining the weight based on the 5th percentile for age.

Additional edits were made throughout the document to be consistent among vaccine studies at MedImmune as described below:

1) Entire document: Solicited adverse events was changed to solicited symptoms.
2) Section 3.1.2 (Exclusion Criteria): Exclusion criterion #6-Infrequent use of over-the-counter medications was specified as treatment for common childhood symptoms and examples of the types of medications allowed were described. For exclusion criteria #8, 9, 14, 15, 17, and 18, the 30-day window after final study vaccine dosing for receipt of blood transfusions or immunoglobulin products, as well as for avoiding close contact with immunocompromised individuals was changed to a 28-day window and "within 28 days after each dose" was added as the allowed window for contact with at risk caregivers. Under history of medical diagnosis (Exclusion criterion #22), bronchoconstriction or treatment with a β_2 agonist (eg, albuterol) was added. In addition, in exclusion criterion #22, the statement "use of mechanical ventilation" was specified for respiratory illness and is described as "excluding elective mechanical ventilation during surgery for subjects in Cohort 1".
3) Section 3.4 (Schedule of Subject Evaluation: 28-34 Days Post Dose 3, Visit 12): Subtitle was changed for clarification, and instructions for final telephone contact were included.
4) Section 3.5.3 (Immune Response Evaluations): Instructions for proper storage and shipment of serum samples was added.
5) Sections 4.2.3 (Notification of Sponsor of Serious Adverse Events) and 4.5 (Other Events Requiring Immediate Reporting): Use of the electronic data capture (EDC) system for reporting SAEs and IREs was removed, as not all data will be captured through this system.
6) Section 4.3 (Safety Management During the Study): A statement was added to specify that SMC decisions regarding discontinuation or resumption of dosing and/or randomization will be communicated promptly to the FDA.

Protocol Version 2.0, [redacted]

All text revisions resulting from this amendment are incorporated in the body of the protocol in Version 2.0. The following major changes to the protocol were made in response to a request from the FDA on [redacted]:

1) The stopping rule regarding MA-LRIs in Section 4.8 of the protocol has been amended as follows: If viral culture results (or, in the event that culture results are not available for technical reasons, PCR results) are not available for a nasal wash specimen collected between 2 days prior to onset through 5 days after onset, an MA-LRI event will be assessed as if associated with shedding of vaccine virus, if the MA-LRI occurs within 28 days post dose.

2) Section 4.1.6 (Study Reporting Period for Enhanced Disease Surveillance): To further clarify the definition of enhanced disease, the last sentence of this section was changed from: "To qualify as an RSV-related hospitalization or death, RSV must be identified in

clinical specimens at the time of illness" to "To qualify as an RSV-related hospitalization or death, either wild-type RSV must have been identified in a clinical specimen OR a valid negative result for RSV testing was not available from a clinical specimen obtained during the window from 2 days prior to onset of symptoms to 5 days after onset of symptoms (i.e. in the absence of a confirmed negative culture, events are analyzed as if associated with shedding of wild-type RSV)". Additional edits were made to this section for clarity.

The following major changes in the protocol were made to reflect changes in the management of dosing interruptions in response to safety events:

1) Section 4.8 (Interruption or Discontinuation of Overall Study Dosing and Randomization-Interruption or Discontinuation of Study Randomization): This section was divided into 2 subsections to separate halt in all dosing from halt in enrollment of new subjects. Items #1 ("Any SAE assessed as related...") and #5 ("...event that contraindicates dosing...") were placed under the subsection titled, "Interruption or Discontinuation of Overall Study Dosing and Randomization" and the items were re-numbered as #1 and #2. Items #2 and 3 were placed under the subsection titled, "Interruption or Discontinuation of Study Randomization" and item #2 was amended to read as follows: "The occurrence of a statistically significant imbalance (analyzed by subject) either cumulatively (ie, among all cohorts) or by cohort in Grade 3 and 4 adverse events (includes MA-LRI events) occurring within 28 days after dosing with more events in vaccine arm compared to placebo arm". Item #4, "The occurrence of a statistically significant imbalance in Grade 2 or higher solicited symptoms..." was eliminated, because solicited symptoms are anticipated reactogenicity events for which imbalance might be anticipated. For clarity, Section 4.8 specifies that "... the IVRS system will be locked and no additional subjects will be randomized into the study until review of the event in question by the Medical Monitor and the SMC," and an additional statement was added at the end of this section stating: "If study randomization has been interrupted pending SMC review of event(s) while other study dosing continues, and if the SMC has found that randomization should not be re-instituted, all study dosing also must be discontinued, unless the SMC has specifically determined that dosing may continue."

The following global changes were made throughout the protocol to reflect changes in study design that are based on medical judgment:

1) Expand the study to global sites and year-round enrollment with 365-day follow-up.

2) Expand the age eligibility criteria for cohort 1 to 5 - <24 months old.

3) Reduce the age limit for restriction of close contacts with an enrolled subject (such as in a household or daycare setting) to < 6 months of age, and similarly to reduce the age limit for restriction of household contacts who are daycare providers or preschool teachers to < 6 months of age.

4) Eliminate 1 of the optional blood draws and to remove a limit of "up to 40 subjects" for the subset of subjects having the optional blood draws, to clarify that there is no given number of subjects required to have optional blood draws.

5) Eliminate the investigational immune response assays for B-cell and T-cell immune responses and to remove the wording, "depending on study site proximity to a testing laboratory" because this only applies to B and T cell assays.

Additional edits were made throughout the document for clarity as described below:

1) Title Page: The title was changed to include children who are <24 months of age and now reads as: A Phase 1/2a, Randomized, Double-Blind, Placebo-Controlled Study to Evaluate the Safety, Tolerability, Immunogenicity, and Viral Shedding of MEDI-559, a Live Attenuated Intranasal Vaccine Against Respiratory Syncytial Virus in Healthy 1 to <24 Month-Old Children. The Study Monitor was changed from MedImmune to [redacted].

2) The Study Abstract was revised to be consistent with changes made within the body of the protocol.

3) Sections 2.1 (Primary Objective) and 2.2 (Secondary Objectives): The primary study period for describing safety and tolerability was clarified as 28 days post last scheduled dose, and a secondary objective was added for clarify as follows: To describe the safety of MEDI-559 through 365 days post dose 1.

4) Section 3.1.2 (Exclusion Criteria): In Exclusion Criterion #6, reference to protocol-specified blood collection was removed to be consistent with Section 4.7 (Interruption or Discontinuation of Study Dosing in Individual Subjects); Day care was specified as full-time in Exclusion Criterion #14; the age limit of close contacts with an enrolled subject was changed from <24 months to < 6 months of age, and the exclusion period was clarified to read, "within 28 days after each dose" in Exclusion Criteria #14 and #18; in Exclusion Criterion #16, the words "for 28 days after each dose" were added to define the time limit that subjects are to avoid close contact with immunocompromised individuals; and Exclusion Criterion #23 was clarified to refer to an "Immediate family member who is an employee of the clinical study site or who is otherwise involved with the conduct of the study".

5) Section 3.1.2 (Exclusion Criteria), Appendix A (Severity Grading Table for Solicited Symptoms and Adverse Events), and Appendix C (Events for Which a Nasal Wash Specimen is Required): Reference to rectal temperature measurement being strongly preferred for accuracy, with temperature measurement by other methods being strongly discouraged was removed.

6) Section 3.2.2 (Blinding): A statement was added regarding MedImmune staff responsible for laboratory assays who have access to potentially unblinded data and the safeguards in place to document that these staff may not unblind other study personnel. Additional information was added regarding interim analyses by cohort with a description of MedImmune staff who must remain blinded during an interim analysis.

7) Section 3.3.5 (Concomitant Medications/Immunizations): Reference to protocol-specified blood collection was removed, to be consistent with Section 4.7 (Interruption or Discontinuation of Study Dosing in Individual Subjects). The currently recommended immunization schedule was clarified as “local guidance (eg, in the USA, the recommendation of the CDC, and the equivalent in other countries)”.

8) Section 3.4 (Schedule of Subject Evaluations): Clarification of the collection period for solicited symptoms was made throughout in this section in order to be consistent with Section 4.1.5 (Study Reporting Period for Solicited symptoms, AEs, MA-LRIs, and SNMCs).

9) Sections 3.5.1 (Safety Evaluations) and 4.1.2 (Definition of Solicited Symptoms): Rectal temperature as the preferred measurement for recording daily temperature was changed to axillary route as the preferred measurement. In the Safety Evaluation Section, the following sentence was removed: “If an axillary temperature of ≥ 99.4°F (≥ 37.4°C) is measured, the temperature should be repeated rectally to confirm the fever”.

10) Section 3.5.2 (Viral Shedding Evaluation-Methods for Sample Collection, Handling, and Testing): The examples given for further characterization of samples that are determined to be positive for RSV and are sent to the MedImmune CTL for further characterization, were clarified to include identification as wild-type or vaccine-like virus.

11) Section 4.6 (Safety Management During the Study): This section was modified to more clearly define the role of the SMC in reviewing blinded and unblinded safety data.

12) Section 4.7 (Interruption or Discontinuation of Study Dosing in Individual Subjects): This section was amended to repeat the exclusion criteria in Section 3.1.2 (Exclusion Criteria), concerning exposure to at risk individuals within 28 days after each dose; and to add the following wording: “at the time of study vaccine administration” to item #1. A statement was added that if Dose 2 is missed, the vaccination window for Dose 3 will be 96 to 120 days post dose 1. In subjects who do not receive 3 doses, a statement was added to clarify the post-dose blood and nasal wash samples that must be obtained when doses are missed. Additionally, for clarification the following statement was added: “For all subjects, unscheduled visits to obtain illness nasal washes must be held in accordance with Appendix C.” Under the subsection titled, “Permanent Discontinuation of Study Vaccine”, a new item #13 was added, which states: “Known or suspected immunodeficiency including HIV”.

13) Section 5.6 (Other Analyses): The last sentence referring to the evaluation of immune responses and immunologic profiling was replaced with the section number where this information is described in detail.

14) Section 7.1 (Ethics and Regulatory Considerations): For clarity, the following wording for maintaining confidentiality was added “or in accordance with local confidentiality rules”.

15) Appendix A (Severity Grading Table for Solicited Symptoms and Adverse Events): The heading row titled, "Adverse Events *without* a Respiratory Component" was changed to "Grading Adverse Event other than MA-LRI," and the heading row titled "Adverse Event *with* a Respiratory Component" was changed to **"**For Grading MA-LRIs**"**

16) Appendix C (Events for Which a Nasal Wash Specimen is Required): The title for the second heading was changed from **"**At Any Time during the Study (Day 0 through the end of RSV season or 180 days after the final dose, whichever is later)" to "At Any Time during the Study (Day 0 through 365 days after randomization)"; and Rales was more clearly defined.
