# Supplementary material for: Safety and Immunogenicity of a Live Attenuated RSV Vaccine in Healthy RSV-Seronegative Children 5 to 24 Months of Age
Source: PLoS One. 2013 Oct 29;8(10):e77104. doi: 10.1371/journal.pone.0077104 (PMC3812203; doi:10.1371/journal.pone.0077104)
Supplement: Sites and IRBs S1 — (DOCX) [file pone.0077104.s003.docx]

**IRBs Names and Addresses**

- Schulman Associates IRB, Inc., Sharon Nelson, M.S.N., R.N., C.N.S., 4445 Lake Forest Drive, Suite 300, Cincinnati, Ohio 45242, Tel. 513-761-4100
  - A. Acevedo, South Miami Clinical Research Group, Miami, FL
  - J. Borders, Central Kentucky Research Associates, Inc, Lexington, KY
  - C. Bowman-Stroud, Four Rivers Clinical Research, Inc., Paducah, KY
  - D. Brandon, California Research Foundation, San Diego, CA
  - M. Cruz, Premier Health Research Center, Downey, CA
  - W. Daly, Bluegrass Clinical Research, Louisville, KY
  - B. Essink, Meridian Clinical Research, LLC, Omaha, NE
  - B. Harvey, Children's Investigational Research Program, Bentonville, AR
  - K. Kim, West Coast Clinical Trials Phase 2-4, LLC, Cypress, CA
  - J. Ley, Holston Medical Group, Kingsport, TN
  - C. Nassim, Nassim, McMonigle, Mescia and Associates, New Albany, IN
  - K. Palanpurwala, Premier Health Research Center, Downey, CA
  - T. Patel, Road Runner Research, LTD, San Antonio, TX
  - P. Qaqundah, Pediatric Care Medical Groups, Inc., Huntington Beach, CA
  - P. Ratner, Sylvana Research, San Antonio, TX
  - E. Reyes, Emmaus Research Center, Inc., Anaheim, CA
  - C. Reyes-Acuna, Intrinsic Research Data, Inc., Corpus Christi, TX
  - M. Simon, Private Practice, Lexington, KY
  - D. Williams, Veritas Research, LLC, Greenville, AL
  - P. Wisman Jr., Pediatric Research of Charlottesville, LLC, Charlottesville, VA
- Metro Health IRB, David Kuentz, D.O., 2500 MetroHealth Drive, Cleveland, OH 44109, Tel. 216-778-2077
  - N. Abughali, MetroHealth Medical Center, Cleveland, OH
- Children's Memorial Hospital IRB, Vita Land, M.D., 2300 Children's Plaza, Box 205, Chicago, IL 60614-3394, Tel. 773-755-6306
- R. Yogev, Children’s Memorial Hospital, Chicago, IL
- Western Institutional Review Board, Theodore D. Schultz, 3535 7th Avenue SW, Olympia, WA 98502, Tel. 360-252-2500
- C. Marchant, Boston University Medical Center, Fall River, MA
- L. Meloy, Virginia Commonwealth University, Richmond, VA
- Connecticut Childrens Medical Center IRB, Francis DiMario, M.D., 282 Washington St., Hartford, CT 06106, Tel. 860-545-9980
- C. Wiley, Connecticut Children’s Medical Center, Hartford, CT
- University of Louisville IRB, Laura Clark, M.D., MedCenter One, Suite 200, 501 E. Broadway, Louisville, KY 40202-1798, Tel.502-852-5188
- K. Bryant, University of Louisville School of Medicine, Louisville, KY
- Memorial Hospital of South Bend IRB, Alicia Dombkowski, 615 North Michigan St., South Bend, IN 46601, Tel. 574-647-3468
- G. Maher, Memorial Medical Group Clinical Research Institute, South Bend, IN
- SUNY Upstate IRB, Stephen Graziano, M.D., 750 East Adams Street, Syracuse, NY 13210, 315-464-4317
- J. Domachowske, SUNY Upstate Medical University, Syracuse, NY
