# Supplementary material for: Safety and Immunogenicity of a Live Attenuated RSV Vaccine in Healthy RSV-Seronegative Children 5 to 24 Months of Age
Source: PLoS One. 2013 Oct 29;8(10):e77104. doi: 10.1371/journal.pone.0077104 (PMC3812203; doi:10.1371/journal.pone.0077104)
Supplement: Study Investigators S1 — (DOCX) [file pone.0077104.s004.docx]

**Study Investigators**

E. Reyes, Emmaus Research Center, Inc., Anaheim, CA

D. Brandon, California Research Foundation, San Diego, CA

W. Daly, Bluegrass Clinical Research, Louisville, KY

K. Palanpurwala, Premier Health Research Center, Downey, CA

C. Marchant, Boston University Medical Center, Fall River, MA

C. Nassim, Nassim, McMonigle, Mescia and Associates, New Albany, IN

J. Domachowske, SUNY Upstate Medical University, Syracuse, NY

J. Borders, Central Kentucky Research Associates, Inc, Lexington, KY

C. Reyes-Acuna, Intrinsic Research Data, Inc., Corpus Christi, TX

K. Bryant, University of Louisville School of Medicine, Louisville, KY

P. Wisman Jr., Pediatric Research of Charlottesville, LLC, Charlottesville, VA

D. Williams, Veritas Research, LLC, Greenville, AL

K. Kim, West Coast Clinical Trials Phase 2-4, LLC, Cypress, CA

M. Cruz, Premier Health Research Center, Downey, CA

B. Harvey, Children's Investigational Research Program, Bentonville, AR

P. Qaqundah, Pediatric Care Medical Groups, Inc., Huntington Beach, CA

G. Maher, Memorial Medical Group Clinical Research Institute, South Bend, IN

B. Essink, Meridian Clinical Research, LLC, Omaha, NE

J. Ley, Holston Medical Group, Kingsport, TN

L. Meloy, Virginia Commonwealth University, Richmond, VA

C. Wiley, Connecticut Children’s Medical Center, Hartford, CT

C. Bowman-Stroud, Four Rivers Clinical Research, Inc., Paducah, KY

T. Patel, Road Runner Research, LTD, San Antonio, TX

M. Simon, Private Practice, Lexington, KY

A. Acevedo, South Miami Clinical Research Group, Miami, FL

P. Ratner, Sylvana Research, San Antonio, TX
