# Supplementary material for: Safety and Immunogenicity of a Live Attenuated RSV Vaccine in Healthy RSV-Seronegative Children 5 to 24 Months of Age
Source: PLoS One. 2013 Oct 29;8(10):e77104. doi: 10.1371/journal.pone.0077104 (PMC3812203; doi:10.1371/journal.pone.0077104)
Supplement: Table S1 — Sequence of the primers and probes for RSV A and B detection. (DOCX) [file pone.0077104.s005.docx]

**Supporting Table 1. Sequence of the primers and probes for RSV A and B detection.**

| **Virus** | **Assay Name** | **Target** | **Sequence** | **Concentration in PCR, nM** |
| --- | --- | --- | --- | --- |
| **RSV A** | RSV-AN-F | N gene | GCTCTTAGCAAAGTCAAGTTGAATGA | 300 |
|  | RSV-AN-R |  | TGCTCCGTTGGATGGTGTATT | 300 |
|  | RSV-AN-P |  | HEX-ACACTCAACAAAGATCAACTTCTGTCATCCAGC | 200 |
| **RSV B** | RSV-BN-F | N gene | GATGGCTCTTAGCAAAGTCAAGTTAA | 300 |
|  | RSV-BN-R |  | TGTCAATATTATCTCCTGTACTACGTTGAA | 300 |
|  | RSV-BN-P |  | FAM-TGATACATTAAATAAGGATCAGCTGCTGTCATCCA | 200 |
| **aDV** | aDV-F |  | AGCAAACCGTGCTGCCTGTAG | 300 |
|  | aDV-R |  | CCCATGCGTACAGCTTCCAT | 300 |
|  | aDV-P |  | HEX-CTTCATCGTGGGGATGTAAAAACCTGG | 200 |
| **SPLV-IPC** | IPC-F |  | GCTATTTGTGCAGACGAATTTGCCT | 300 |
|  | IPC-R |  | CGTAGCAAGAGAACTGCACGAGGAT | 300 |
|  | IPC-P |  | FAM-CGAACTGCACCAACACATGGAGGTGAGG | 200 |

aDV, armored dengue virus, present as a spiked recovery control from viral nucleic acid isolation; mSPLV-IPC, sweet potato leaf virus internal positive control spiked into RT-PCR reactions ; PCR, polymerase chain reaction; RSV, respiratory syncytial virus
