# Supplementary material for: Safety and Immunogenicity of a Live Attenuated RSV Vaccine in Healthy RSV-Seronegative Children 5 to 24 Months of Age
Source: PLoS One. 2013 Oct 29;8(10):e77104. doi: 10.1371/journal.pone.0077104 (PMC3812203; doi:10.1371/journal.pone.0077104)
Supplement: Table S2 — RT-PCR conditions. (DOCX) [file pone.0077104.s006.docx]

**Supporting Table 2. RT-PCR conditions.**

| **Step** | **Temperature, °C** | **Time, min** | **Repeat** |
| --- | --- | --- | --- |
| Reverse transcription | 50 | 15 | None |
| Activation of Taq polymerase | 95 | 2 | None |
| Denaturation of DNA template | 95 | 0.5 | 40 cycles |
| Annealing of primers/probe to target and extension of primers | 60 | 1 |  |

RT-PCR, reverse transcriptase polymerase chain reaction.
