# Supplementary material for: Safety and Immunogenicity of a Live Attenuated RSV Vaccine in Healthy RSV-Seronegative Children 5 to 24 Months of Age
Source: PLoS One. 2013 Oct 29;8(10):e77104. doi: 10.1371/journal.pone.0077104 (PMC3812203; doi:10.1371/journal.pone.0077104)
Supplement: Table S3 — Wild-type RSV/MEDI-559 ΔSH assay primer sequences. (DOCX) [file pone.0077104.s007.docx]

**Supporting Table 3. Wild-type RSV/MEDI-559 ΔSH assay primer sequences.**

| **Name** | **Primer ID** | **Sequence** |
| --- | --- | --- |
| aDV | F2 | 5-GAAAGCCACGGTTTGAGCAAA-3' |
| aDV | R2 | 5'-GTTCTGTGCCTGGAATGATGCT-3' |
| RSV ΔSH | F4 | 5'-CCATCACAATCACAAACACTCTGTGG-3 |
| RSV ΔSH | R4 | 5'-GACTCCTGGTGTTGTTGAAGCTAGTATG-3' |

aDV, armored dengue virus; ΔSH, small hydrophobic gene deletion; RSV, respiratory syncytial virus
