# Supplementary material for: Safety and Immunogenicity of a Live Attenuated RSV Vaccine in Healthy RSV-Seronegative Children 5 to 24 Months of Age
Source: PLoS One. 2013 Oct 29;8(10):e77104. doi: 10.1371/journal.pone.0077104 (PMC3812203; doi:10.1371/journal.pone.0077104)
Supplement: Table S4 — Wild-type RSV/MEDI-559 ΔSH assay RT-PCR condition. (DOCX) [file pone.0077104.s008.docx]

**Supporting Table 4. Wild-type RSV/MEDI-559 ΔSH assay RT-PCR condition.**

| **Step** | **Condition** |
| --- | --- |
| Reverse transcription | 15 min, 50°C |
| Activation of Taq polymerase | 2 min, 94°C |
| PCR cycle (40 cycles) | 15 s, 94°C |
|  | 30 s, 60°C |
|  | 1 min, 68°C |
| Taq polymerase elongation | 5 min, 68°C |
| Storage | 4°C |

RT-PCR, reverse transcriptase polymerase chain reaction
