# Supplementary material for: Safety and Immunogenicity of a Live Attenuated RSV Vaccine in Healthy RSV-Seronegative Children 5 to 24 Months of Age
Source: PLoS One. 2013 Oct 29;8(10):e77104. doi: 10.1371/journal.pone.0077104 (PMC3812203; doi:10.1371/journal.pone.0077104)
Supplement: Table S5 — Sequence of the primers and probes for respiratory virus detection. (DOCX) [file pone.0077104.s009.docx]

**Supporting Table 5. Sequence of the primers and probes for respiratory virus detection.**

| **Virus** | **Assay Name** | **Target** | **Sequence** | **nM** |
| --- | --- | --- | --- | --- |
| **Flu A** | Flu-A-M-F | M segment | TGGCCAGCACTACAGCTAAGG | 300 |
|  | Flu-A-M-R |  | CCATGGCCTCTGCTGCTT | 300 |
|  | Flu-A-M-P |  | HEX-TCACTCGATCCAGCCATTTGCTCCAT | 200 |
| **Flu B** | Flu-BHA-F | HA segment | GTGGATTAAACAAAAGCAAGCCTTAC | 300 |
|  | Flu-BHA-R |  | GGAGGTCTATATTTGGTTCCATTG G | 300 |
|  | Flu-BHA-P |  | FAM-CCATAGGAAATTGCCCAA-MGB | 200 |
| **RSV A** | RSV-AN-F | N gene | GCTCTTAGCAAAGTCAAGTTGAATGA | 300 |
|  | RSV-AN-R |  | TGCTCCGTTGGATGGTGTATT | 300 |
|  | RSV-AN-P |  | HEX-ACACTCAACAAAGATCAACTTCTGTCATCCAGC | 200 |
| **RSV B** | RSV-BN-F | N gene | GATGGCTCTTAGCAAAGTCAAGTTAA | 300 |
|  | RSV-BN-R |  | TGTCAATATTATCTCCTGTACTACGTTGAA | 300 |
|  | RSV-BN-P |  | FAM-TGATACATTAAATAAGGATCAGCTGCTGTCATCCA | 200 |
| **PIV1** | hPIV1-HN-F | HN gene | GTTGTCAATGTCTTAATTCGTATCAATAATT | 900 |
|  | hPIV1-HN-R |  | GTAGCCTMCCTTCGGCACCTAA | 900 |
|  | hPIV1-HN-P |  | HEX-TAGGCCAAAGATTGTTGTCGAGACTATTCCAA | 200 |
| **PIV2** | hPIV2-HN-F | HN gene | GCATTTCCAATCTTCAGGACTATGA | 900 |
|  | hPIV2-HN-R |  | ACCTCCTGGTATAGCAGTGACTGAAC | 900 |
|  | hPIV2-HN-P |  | FAM-CCATTTACCTAAGTGATGGAATCAATCGCAAA | 200 |
| **hPIV3** | hPIV3-P-F | NP gene | CATGATTGACCCAATCTGATCCACTGTG | 300 |
|  | hPIV3-P-R |  | GCTTTTTCACCCGATTGGAAGCTTTCA | 300 |
|  | hPIV3-P-P |  | FAM-TACCAGCCCTGCCTGCACTGTTCCATCTTG | 200 |
| **HMPV** | HMPV-N-F | N gene | CATATAAGCATGCTATATTAAAAGAGTCTC | 200 |
|  | HMPV-N-R |  | CCTATTTCTGCAGCATATTTGTAATCAG | 200 |
|  | HMPV-N-P |  | FAM-TGYAATGATGAGGGTGTCACTGCGGTTG | 50 |

HMPV, human metapneumovirus; hPIV, human parainfluenza virus; PIV, parainfluenza virus; RSV, respiratory syncytial virus
