# Supplementary material for: Safety and Immunogenicity of a Live Attenuated RSV Vaccine in Healthy RSV-Seronegative Children 5 to 24 Months of Age
Source: PLoS One. 2013 Oct 29;8(10):e77104. doi: 10.1371/journal.pone.0077104 (PMC3812203; doi:10.1371/journal.pone.0077104)
Supplement: Text S1 — RSV qRT-PCR. (DOCX) [file pone.0077104.s010.docx]

**Supporting Text 1. RSV qRT-PCR.**

This RSV A/B assay was developed by MedImmune, LLC, to determine the presence of MEDI-559, RSV A, or RSV B virus and employed to evaluate the shedding of vaccine virus or wild-type RSV following vaccination or natural infection. The assay uses a quantitative polymerase chain reaction (qPCR) platform; however, the results are reported as either positive or negative. Thus, this assay is a qualitative assay.

RNA from a nasal wash sample is isolated using the Roche HighPure nucleic acid extraction kit, using a sample lysis buffer spiked with an RNA-containing virus-like particle (armored dengue virus [aDV]) as RNA isolation control. The isolated RNA is subjected to a single-tube reverse transcriptase qPCR [qRT-PCR]. Two separate multiplex qRT-PCR reactions are performed. The first reaction contains primers and TaqMan probes (Supplementary Table 1) for the detection of RSV A and RSV B. The second reaction contains primers and TaqMan probes for the amplifications of the aDV RNA and of an internal positive control of RNA from sweet potato leaf virus, which is spiked into the RT-PCR mastermix. The adequacy of the RNA isolation of samples is monitored by the amplification of the aDV amplicon, whereas amplification of the internal positive control RNA provides a separate check on the presence or absence of PCR inhibitors. The RT-PCR condition is listed in Supplementary Table 2.
