# Supplementary material for: Safety and Immunogenicity of a Live Attenuated RSV Vaccine in Healthy RSV-Seronegative Children 5 to 24 Months of Age
Source: PLoS One. 2013 Oct 29;8(10):e77104. doi: 10.1371/journal.pone.0077104 (PMC3812203; doi:10.1371/journal.pone.0077104)
Supplement: Text S2 — Wild-type RSV/MEDI-559 ΔSH assay. (DOCX) [file pone.0077104.s011.docx]

**Supporting Text 2. Wild-type RSV/MEDI-559 ΔSH assay.**

This RT-PCR assay was developed and qualified to detect and differentiate MEDI-559 vaccine virus from wild-type RSV A and was employed to evaluate the shedding of vaccine or wild-type RSV A following vaccination or natural infection. The RNA is subjected to RT-PCR in a single reaction, using primers (see Supplementary Table 3) binding to the flanking regions of the small hydrophobic (SH) gene deletion in MEDI-559 virus. RT-PCR amplification (see Supplementary Table 4) of MEDI-559 produces an amplicon of 541 base pairs (bp), whereas wild-type RSV A, which does not have the SH deletion, will yield an amplicon of 961 bp. The amplicons generated by RT-PCR are detected using a microfluidic electrophoresis instrument (the Agilent BioAnalyzer). RNA isolation from the samples is monitored by inclusion of an RNA-containing virus-like particle (aDV) in the sample lysis buffer. The adequacy of RNA isolation and absence of PCR inhibitors in the sample is checked by RT-PCR and detection of the aDV amplicon.
