# Supplementary material for: Safety and Immunogenicity of a Live Attenuated RSV Vaccine in Healthy RSV-Seronegative Children 5 to 24 Months of Age
Source: PLoS One. 2013 Oct 29;8(10):e77104. doi: 10.1371/journal.pone.0077104 (PMC3812203; doi:10.1371/journal.pone.0077104)
Supplement: Text S3 — Respiratory virus detection assays. (DOCX) [file pone.0077104.s012.docx]

**Supporting Text 3. Respiratory virus detection assays.**

This assay was developed to determine the presence of absence of common respiratory viruses in nasal wash and employed to evaluate the shedding of various common viruses by subjects enrolled in clinical trials following vaccination or natural infection. The assay uses a quantitative PCR format; however, the results are reported as either positive or negative. Thus, the assay is a qualitative assay.

The isolated RNA is subjected to 5 separate multiplex qRT-PCR. Each multiplex qRT-PCR assay contains the primers and TaqMan probes (see Supplementary Table 5) to detect 2 different targets, comprising 8 human virus targets and 2 control RNAs, aDV and an IPC. The assays are grouped as follows: influenza A/influenza B; RSV A/RSV B; parainfluenza virus 1 (PIV1)/PIV2; PIV3/aDV; and human metapneumovirus (HMPV)/IPC. The RT-PCR condition is in Supplementary Table 2.
